# Overexpression of *WsSGTL1* Gene of *Withania somnifera* Enhances Salt Tolerance, Heat Tolerance and Cold Acclimation Ability in Transgenic *Arabidopsis* Plants

**DOI:** 10.1371/journal.pone.0063064

**Published:** 2013-04-30

**Authors:** Manoj K. Mishra, Pankaj Chaturvedi, Ruchi Singh, Gaurav Singh, Lokendra K. Sharma, Vibha Pandey, Nishi Kumari, Pratibha Misra

**Affiliations:** 1 Council of Scientific and Industrial Research - National Botanical Research Institute, Rana Pratap Marg, Lucknow, Uttar Pradesh, India; 2 Sanjay Gandhi Post Graduate Institute of Medical Sciences, Lucknow, Uttar Pradesh, India; 3 Banaras Hindu University, Varanasi, Uttar Pradesh, India; RIKEN Plant Science Center, Japan

## Abstract

**Background:**

Sterol glycosyltrnasferases (SGT) are enzymes that glycosylate sterols which play important role in plant adaptation to stress and are medicinally important in plants like *Withania somnifera.* The present study aims to find the role of *WsSGTL1* which is a sterol glycosyltransferase from *W. somnifera,* in plant’s adaptation to abiotic stress.

**Methodology:**

The *WsSGTL1* gene was transformed in *Arabidopsis thaliana* through *Agrobacterium* mediated transformation, using the binary vector pBI121, by floral dip method. The phenotypic and physiological parameters like germination, root length, shoot weight, relative electrolyte conductivity, MDA content, SOD levels, relative electrolyte leakage and chlorophyll measurements were compared between transgenic and wild type *Arabidopsis* plants under different abiotic stresses - salt, heat and cold. Biochemical analysis was done by HPLC-TLC and radiolabelled enzyme assay. The promoter of the *WsSGTL1* gene was cloned by using Genome Walker kit (Clontech, USA) and the 3D structures were predicted by using Discovery Studio Ver. 2.5.

**Results:**

The *WsSGTL1* transgenic plants were confirmed to be single copy by Southern and homozygous by segregation analysis. As compared to WT, the transgenic plants showed better germination, salt tolerance, heat and cold tolerance. The level of the transgene *WsSGTL1* was elevated in heat, cold and salt stress along with other marker genes such as *HSP70, HSP90*, *RD29, SOS3* and *LEA4-5.* Biochemical analysis showed the formation of sterol glycosides and increase in enzyme activity. When the promoter of *WsSGTL1* gene was cloned from *W. somnifera* and sequenced, it contained stress responsive elements. Bioinformatics analysis of the 3D structure of the *WsSGTL1* protein showed functional similarity with sterol glycosyltransferase *AtSGT* of *A. thaliana.*

**Conclusions:**

Transformation of *WsSGTL1* gene in *A. thaliana* conferred abiotic stress tolerance. The promoter of the gene in *W.somnifera* was found to have stress responsive elements. The 3D structure showed functional similarity with sterol glycosyltransferases.

## Introduction

In *Withania somnifera,* withanolides are structurally diversified by means of glycosylation and glycosylated withanolides are termed as withanosides or glycowithanolides [1] which are present in roots [2], [3] and leaves [4]. Withanosides have been reported to possess immunomodulatory, adaptogenic, anticonvulsant, neurogenerative and anti-oxidative properties [5], [6]. In some recent reviews natural withanolides have been discussed in detail in regard to their occurrence in different plant genera, and their diverse biological activities [7], [8]. Withanosides are steroidal lactones with one or more glucose units attached to C-3 or C-27 positions due to glycosylation which changes the activity of these molecules and hence there is a change in their metabolic participation. Sterol glycosyltransferases (SGTs) are the enzymes which catalyze the transfer of sugar molecules on to acceptor sterol molecules from activated sugar donor such as UDP-glucose. The effect of sugar positions in ginsenosides with sugar moieties attached only to the C-3 position of the steroid-like structure and their inhibitory potency on Na+/K+-ATPase activity for cardiac therapy has been tested in *Panax ginseng* by Chen et al. [9]. *SGT*s play an important role in metabolic plasticity during adaptive responses. The glycosyltransferase multigene family is categorized into 94 numbered families according to sequence similarity, signature motifs, stereochemistry of the glucoside linkage formed, and known target specificity (http://afmb.cnrs-mrs.fr/CAZY). Of these 94 families, *SGT*s have been grouped into Family 1 of the classification scheme.

*SGT*s, that glycosylate steroidal hormone, such as brassinosteroids, function as growth and development regulators in plants [10], [11], [12]. Brassinosteroids molecules, due to glysosylation, become inaccessible to the plant resulting in slowing down of growth and development. Role of secondary metabolites and brassinosteroids in plant defense against biotic and abiotic stresses has been published in detail in a recent review by Bartwal et al. [13]. The differential responses to biotic and abiotic stresses and the transcriptional level of effector genes from secondary metabolism have been discussed in detail by Glombitza et al. [14]. *SGT*s in plants are involved in changed sensitivity to stress hormones and changed tolerance to biotic and abiotic stresses [15], [1].

From *W. somnifera,* a novel sterol glycosyltransferases specific to a 27-β hydroxyl steroidal lactone [16] and another sterol glycosyltransferase for 3-β hydroxy position [17] were identified in our laboratory. Additionally, one member of the SGT family (*WsSGTL1*) encoding 3- β hydroxy glycosyltransferase was cloned and characterized (Gen-Bank accession number DQ356887) [15]. Recently, we have identified 3 more members of *SGT* gene family from *W. somnifera* through RACE (Rapid Amplification of cDNA Ends) and characterized them in different stress conditions [1]. The increase in the level of expression of these *SGT*s under heat and cold stress indicated the role of sterol modifications in abiotic stress [1] which needs to be explored further. The 3D structures of *WsSGTL1* and *AtSGT* (*SGT* from *Arabidopsis*) contained sterol binding and UDP-sugar binding domains and were superimposable.

In the present study, *WsSGTL1* was transformed in *Arabidopsis* to investigate the role of this gene in plant’s response to salt, heat and cold stress and to analyze the resultant phenotypic and physiological changes. The behavior of the transgene along with other stress responsive genes was analyzed using semi quantitative RT-PCR and quantitative real time PCR. *WsSGTL1* promoter analysis showed potential *cis*-acting elements for response to salt, heat and cold stress suggesting the regulation of this gene during stress in *W.somnifera*.

## Materials and Methods

### Transgenic Plant Generation

**Figure 1:**
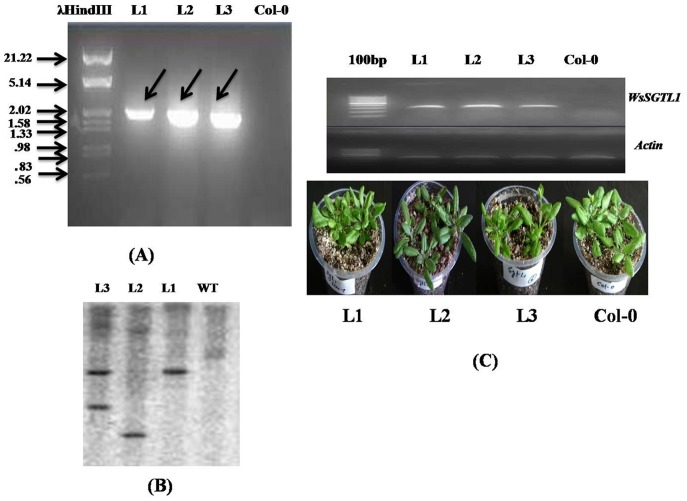
Molecular characterization and phenotype of *WsSGTL1* over expressing transgenic *Arabidopsis* plants. PCR analysis of three positive transgenic lines of *A. thaliana* by Gene Specific Primers in T_3_ generation (amplicon size 2.1 Kb) (**A**). Three transgenic lines were confirmed by Southern analysis. From Rt to Lt, Lane 1-WT, Lane 2-L1, Lane 3-L2 and Lane 4-L3, were selected for all the experiments (The probes were 700 bp and designed from the 5′ unconserved region of the *WsSGTL1*) (**B**). Semi quantitative RT-PCR analysis of two-week-old WT and independent *35S-WsSGTL1* transgenic lines and morphological comparisons of three-week-old WT and *WsSGTL1* over-expressing lines under normal growth conditions (**C**).

Full-length *WsSGTL1* gene sequence was amplified from cDNA using *WsSGTL1*F1 and *WsSGTL1*R1 primers (Table S1). The cDNA was synthesized using the Revert AID First Strand cDNA synthesis kit (Fermentas) from RNA isolated from young leaves of *W.somnifera*, using the Spectrum Plant Total RNA kit (SIGMA). The PCR product was ligated into the binary vector pBI121 (Clonetech) at *Xba1* and *SacI* sites (Figure S1). The plasmid was transformed into *Agrobacterium tumefaciens* (GV3101 strain; Figure S2) and further used to transform *A. thaliana* ecotype Col-0 (wildtype, WT) by floral dip method [18]. Transgenic lines were screened on ½ MS medium containing 50 µg ml^−1^ kanamycin [19]. Transgenic lines selected on kanamycin were further screened by PCR analysis (Figure 1A; Figure S3). They were confirmed to be single copy by Southern (Figure 1B) and homozygous by segregation analysis up to T3 generation. Genomic DNA was isolated by C-TAB method. Ten micrograms of DNA from each sample were digested with *EcoR1* restriction endonuclease, separated on a 1% agarose gel and transferred to Hybond-N+ membranes (Sigma Chemical Company, St. Louis, MO). DNA blot hybridization was performed using a ^32^P-labeled 700 bp of 5′ prime unconserverd region of *WsSGTL1* gene as a probe. *WsSGTL1* gene expression was analyzed by semi-quantitative PCR (Figure 1C). For further study, three independent transgenic lines L1, L2 and L3 were selected. For each experiment, three biological and two technical replicates were used.

### Biochemical Analysis by HPLC-TLC and Enzyme Assay

Wild type as well as all the three transgenic *Arabidopsis* lines was used for characterization of sterols. Briefly, 100 mg (DW) of plant material was extracted in acetone overnight at room temperature with brief agitation. The resulting homogenous extract was then filtered through 0.2 µM filter (Millipore, USA). Acetone was evaporated and dried extract was dissolved in 100% methanol before quantitative analysis of sterols by HPLC-Prominence Diode Array (HPLC-PDA). C18 column of Merk Purospher star® (250 × 4.6 mm, 5 µm pore size) protected with guard column of same chemistry attached to Shimadzu (Japan) LC-10 system controller comprising an LC-10AT dual pump system, an SPD-M20A PDA Detector and rheodyne injection valve furnished with a 20 µL sample loop were used in HPLC-PDA. Elution of sterols was carried out at a flow rate of 2 ml/min with isocratic solution of acetonitrile and water (95∶5 v/v). Quantification of sterols was performed at 202 nm, 34°C. The eluted fractions were spotted on TLC and run using methanol: chloroform (1.5∶8.5, v/v) as the mobile phase. The plates were developed with vanillin sulfuric acid (1%, w/v in 50% sulfuric acid) as spray reagent and chromogenically visualized after heating at 110°C. ß-Sitosterol and ß-sitosterol glycoside procured from sigma were used as standards. The enzyme activity was checked in crude extracts of transgenic plants using the radiolabelled (UDP-C^14^-glucose) enzyme assay described previously [1]. Sitosterol and stigmasterol were used as substrates for enzyme assays.

### Salt Stress Treatment for *A. thaliana*

Seeds of WT and *WsSGTL1* transgenic plants (lines L1, L2 and L3) of *A. thaliana* were germinated on ½ MS media with 2% sucrose and 0.85% agar in a controlled culture room set at 22°C under long day (LD) conditions (16 h light and 8 h dark) with white light illumination (120 µmol/m^2^ s^−1^) provided by fluorescent tubes. For providing salt stress, NaCl was added to the medium at 50, 100 and 150 mM concentrations, while water (0 mM NaCl) was taken as a control. The observed parameters were: 1) germination percentage, 2) root length, 3) shoot length and 4) survival. Transcript level of *WsSGTL1* was checked by real time PCR under normal conditions in 14 days old transgenic plants grown on ½ MS at 0 (water), 50, 100 and 150 mM NaCl. The expression of *LEA4-5* and *SOS3* gene as a marker of salt stress was determined through semi quantitative PCR under 100 mM NaCl stress after 24 h. After salt stress the relative electrolyte conductivity (REC) was measured for both WT and transgenic plants to assess stress adaptation.

### Heat Stress Treatment for WT and Transgenic *A. thaliana* and Lipid Peroxidation Assay

For phenotypic observation under heat stress, 7-days-old and 14-day-old seedlings of WT and transgenic *A. thaliana* were grown on Petriplates, whereas, 21-days-old plants were examined in the pots. The procedure reported by Larkindale et al. [20] was followed except that the seedlings were given heat stress of 42°C instead of 45°C, directly in the incubator for 4 h and seedlings were allowed to recover for 5 days under 16 h light/8 h dark cycle at 22°C. All the heat treatments were performed in the dark. For quantitative real time and semi quantitative PCR analysis, WT and transgenic *A. thaliana* plants (lines L1, L2 and L3) were grown for 14 days under 16 h light/8 h dark and the treatment was given at 42°C for 4 h. Leaves after the treatment, were harvested immediately and stored at −80°C. The expression of *HSP70* and *HSP90* as a marker of heat shock gene was determined through semi quantitative PCR at 4 h and the expression of *WsSGTL1* was deteremined by quantitative real time PCR. Analysis of lipid peroxidation assay by malondialdehyde (MDA) content which is a measure of heat stress was performed as described by the protocol by Larkindale et al. [20]. In short, plants grown on agar plates in light for 14 days were heated to 38°C for 90 min, cooled to room temperature for 120 min and again heated to 42°C for 180 min. This was termed as acquired thermo tolerance. Seedlings were heated to 42°C for 60 min and left to recover under normal light conditions for 2 days. This was termed as basal thermo tolerance. Leaf tissue (100 mg) was homogenized by adding 0.5 ml 0.1% (w/v) trichloroacetic acid (TCA). Homogenate was centrifuged for 10 min (15000 g at 4°C) and the supernatant was collected. The 0.5 ml of supernatant was mixed with 1.5 ml of 0.5% thiobarbituric acid (TBA), diluted in 20% TCA, and was incubated in water bath at 95°C for 25 min. Finally, it was incubated on ice to complete the reaction. The absorbance was measured at 532 and 600 nm. The values of OD_600_ were subtracted from the MDA-TBA complex values at 532 nm and MDA concentration was calculated using the Lambert-Beer law with an extinction coefficient €^M^ = 155 mM^−1 ^cm^−1^. Each sample was compared to that of WT (Col-0) taken as control. Results were averaged over three separate experiments.

### Cold Stress and Determination of Freezing Tolerance by Relative Electrolyte Leakage

For cold stress, both WT and transgenic lines of *A. thaliana* (lines L1, L2 and L3) were grown for 14 days in controlled chamber at 22°C under constant light (100 µmol m^−2^ s^−1^) and cold treatment was given at 4°C for 24 h. The expression of *WsSGTL1* was estimated by real time PCR. The expression of *RD29a* and *RD29b,* cold responsive genes, were determined through semi quantitative PCR at 24 h. Monitoring of plant survival by whole plant freezing test based on the protocol of Xin and Browse [21] was performed separately with the seedlings grown on petriplates. Assessment of injury (and the freezing tolerance) was done by measuring electrolyte leakage from freeze-thaw injured tissues as described by Verslues [22]. The non-acclimated (NA; just prior to administering cold acclimation), and the cold acclimated (CA; plants survived after cold stress) WT and transgenic plants were placed in test tubes in a chiller bath for 1.5 h at −1°C in a completely randomized design, after which ice chips were added to initiate ice-nucleation. After an additional hour of incubation at −1°C, temperature was lowered at a rate of 1°C per hour until it reached −10°C for both NA and CA plants. Frozen samples (three replicates) of each set were removed from the chiller bath and thawed on ice overnight followed by incubation in 20 ml distilled water, vacuum infiltration (3 times for 3 min each at 25 psi), and shaking for 1 h at 250 rpm. Electrical conductivity was measured for each sample with a conductivity meter before and after autoclaving (121°C for 20 min). Initial leakage was expressed as percent of the final conductivity and the percent leakage for each treatment temperature was converted to percent injury. The temperature, at which 50% injury occurred (LT_50_), was defined as the freezing tolerance [23].

### Measurements of Oxidative Stress - SOD Enzyme Activity and Relative Electrolytic Conductivity

Total amount of superoxide dismutase (SOD) enzyme is estimated as a function of nitro blue tetrazolium (NBT) reduction using spectrophotometer. Leaf samples (250 mg) from the seedlings were homogenized in a pre cooled mortar in homogenizing buffer containing 0.1 mM EDTA, 0.5% (v/v) Triton-X 100 and 1% (w/v) polyvinylpyrrolidone (PVP) in 100 mM phosphate buffer (pH 7.8). The homogenate was transferred to 1.5 ml Eppendorf tubes and centrifuged at 13,000 rpm for 20 min at 4°C. The supernatant was collected and total SOD levels were estimated according to Beyer and Fridovich [24]. Protein content was estimated according to the dye binding method of Bradford [25]. The total SOD activity was measured by adding 20 µl supernatant to a reaction mixture containing 4.4% (w/v) Riboflavin, 57 µm NBT, 10 mM L-Methionine and 0.025% (v/v) Triton-X 100 in 100 mM phosphate buffer. One unit of enzyme activity was defined as the amount of enzyme required for 50% inhibition of NBT reduction in 2 min at 25°C.

### Chlorophyll Measurements for Finding the Effect of Transgene

An Imaging-PAM, M-Series Chlorophyll Fluorometer (Walz, Effeltrich, Germany) was used to study the chlorophyll fluorescence parameters. Calculations of various chlorophyll ﬂuorescence parameters were done as given in Maxwell and Johnson [26]. The maximum photochemical efficiency of photosystem II (PSII), (Fv/Fm: where Fm is maximum fluorescence of the dark-adapted leaf under a light saturating flash and Fv is maximum variable fluorescence, Fm *–* F0) was measured on leaves after 20 min of dark adaptation. The effective quantum yield of PSII (Y) was calculated as (Fm-Fs)/Fm. Non photochemical quenching (NPQ) is a measurement that indicates a change in efficiency of excess excitation energy dissipation by heat. NPQ collectively indicates heat dissipation triggered by low thylakoid lumen pH, state transitions of PSII centers, and photo inhibition. Y (NPQ) is a measure of the fraction of photons absorbed by PSII antennae. Increased NPQY is an indication of protective strategies at PSII. Y (NO) is the fraction of photons dissipated by dissociation of light-harvesting complex II and indicates irreversible PSII damage.

### Identification of *WsSGTL1* Promoter Sequence

The promoter sequence of *WsSGTL1* was determined by genome walking method using universal Genome Walker kit (Clontech, USA). Gene specific primers were designed from the known cDNA sequence of *WsSGTL1* to move upstream of gene in genomic DNA. Genomic DNA was digested with four different restriction enzymes (*EcoR*V, *Dra*I, *Pvu*II and *Ssp*I), ligated with adaptors and PCR was performed using Advantage 2 polymerase mix (Figure S4A).

### Bioinformatics Analysis of *WsSGTL1* and *AtSGT* for Functional Similarity

**Figure 2:**
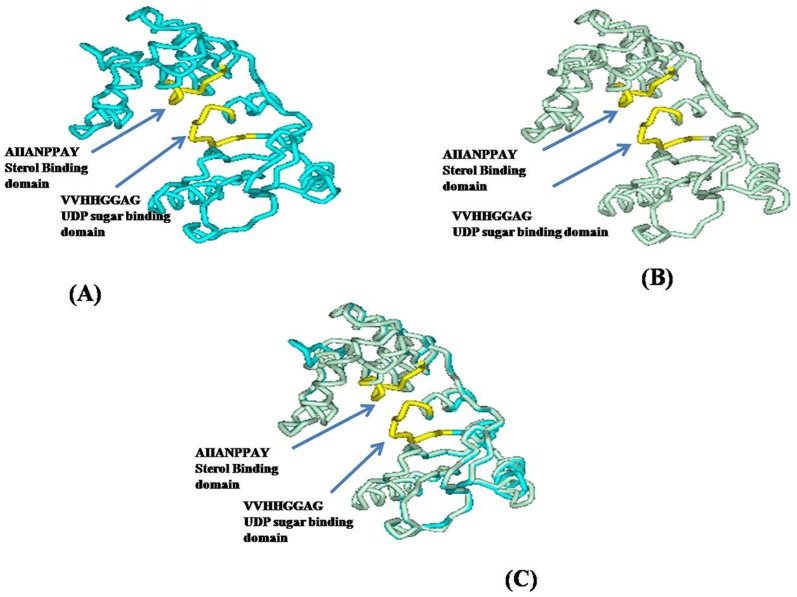
Structural comparison between WsSGTL1 and AtSGT protein. Three-dimensional model of the WsSGTL1 and AtSGT protein as constructed by Phyre2 server using the backbone ‘C3hbjA’. 3D model of WsSGTL1 with sugar binding domain and sterol binding domain (**A**). 3D model of AtSGT with sugar binding domain and sterol binding domain highly similar to WsSGTL1 protein structure (**B**). Structural similarity by superimposition of WsSGTL1 and AtSGT (1.423 Å) (**C**).

Open reading frames (ORFs) of both genes were identified using ORF finder (NCBI), and the protein sequence deduced from the DNA sequence. Homology comparison was carried out by BLASTN and BLASTP (http://www.ncbi.nlm.nih.gov/Blast.cgi). Preliminary properties of the encoded protein were predicted by ProtParam [27]. The pdb file was generated by Phyre server2 with 99% confidence and 99% coverage to the backbone 28. The 3D model was developed by Discovery studio 2.5 using the same backbone of ID **‘c3hbjA’** (Figures 2A–C; Figures S5A–C). ‘C3hbjA’ is a 3D model of sterol glycosytransferase protein available at the online servers.

## Results

### Effect of Salt Stress on WT and Transgenic *A. thaliana* Plants

**Figure 3:**
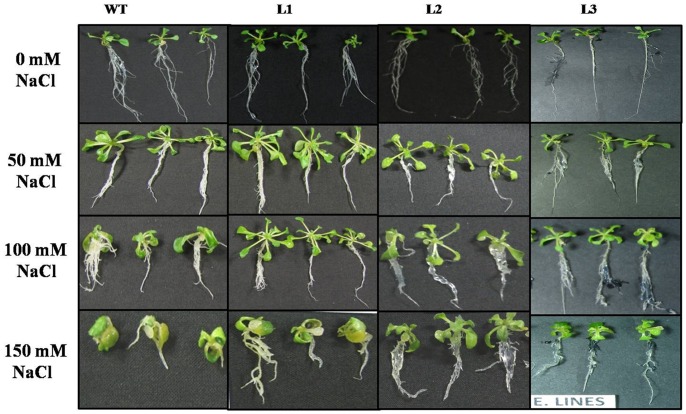
Phenotypic difference between WT (Col-0) plants and *WsSGTL1* overexpression lines of *A.* thaliana. Comparison of shoot, root and leaf growth between *WsSGTL1* lines (L1, L2 and L3) and WT plants grown on MS medium (**A**)**.** With 50 mM NaCl (**B**)**.** With 100 mM NaCl (**C**)**.** With 150 mM NaCl (**D**)**.** photographs were taken after 14 days of germination.

**Figure 4:**
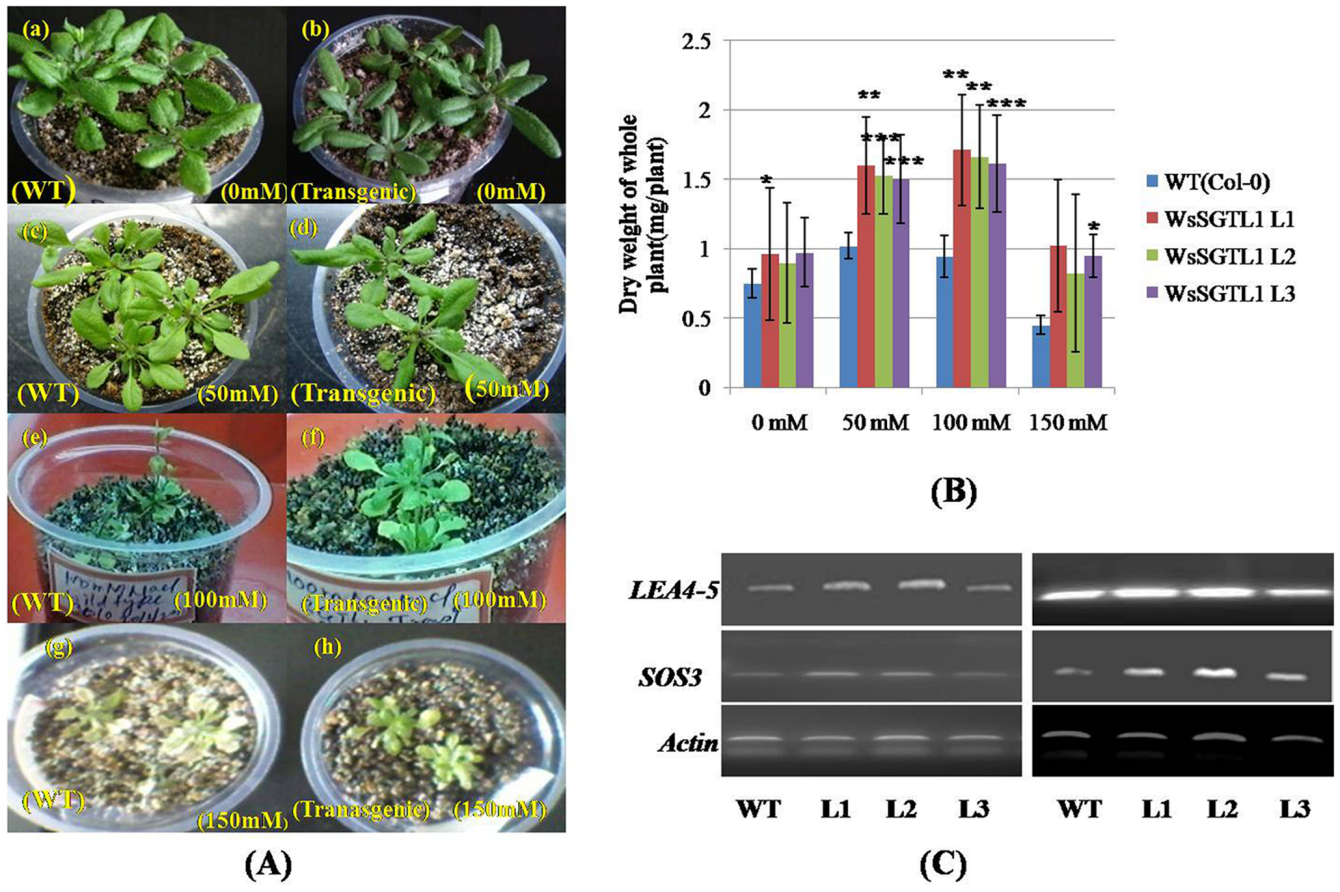
Phenotypes and salt tolerance of the transgenic plants. Salt stressed 3-weeks-old soil grown plants, irrigated with the indicated NaCl solutions every second day up to 14 days. Phenotypes of plants after 14 d of treatment (**A**)**.** Dry weight of whole plants measured after 14 days of salt stress. Values are mean ± SE, n = 10, (*) for *P≤0.05*, (**) for *P≤0.01,* (***) for *P≤0.001* or *0.005,* significantly different from the control (t-test) (**B**)**.** Two-weeks-old seedlings of WT and transgenic lines were used for RNA extraction. To provide salt stress, seedlings were treated with 100 mM NaCl for 24 h before RNA isolation. The transcript level of two stress genes was determined by RT-PCR analyses. The stress genes used for the tests were late embryogenesis abundant proteins *LEA4-5* and Salt overlay sensitive gene *SOS3* (AF192886) (**C**)**.**

We evaluated the phenotypic and physiological changes that occurred in WT and transgenic plants (lines L1, L2 and L3) of *A. thaliana* under salt stress. Both WT and *WsSGTL1* transgenic lines of *A. thaliana* germinated well at 50 and 100 mM NaCl (Figures S6A–C; Figure S7). At higher concentration of NaCl (150 mM), there was poor germination of WT seeds, as compared to the transgenic seeds (Figure S6D; Figure S7). Germination percentage was calculated as number of seeds germinated per sixty seeds. After germination, only transgenic lines grew well. Also, at 100 mM of NaCl the shoot length and root length of transgenic plants was more *(P≤0.001)* as compared to WT plants (Col-0) (Figure 3). The seedlings were transferred to pots and allowed to grow on soil for 3 weeks to analyze the salt response. The plants were then irrigated every second day for 14 d with water containing NaCl (50, 100 or 150 mM, Figure 4A). WT plants showed wilting symptoms after 7 d of treatment of 100 and 150 mM NaCl, while the transgenic lines remained green even after 14 d of similar treatments. The survival data of the salt stressed plants has been shown in Table 1. Similarly, the fresh weight/dry weight (both fresh weight and dry weight were measured to ascertain the biomass) and number of true leaves were more in transgenic *(P≤0.01)* than WT (Figures 3, 4B; Figure S8) plants. The expression of *LEA4-5* and *SOS3* (salt overlay sensitive) gene as determined by semi quantitative PCR was significantly increased in all lines under 100 mM salt stress (Figure 4C).

**Table 1:** Survival of plants under salinity conditions in the soil based analysis platform.

Inflorescence height (cm)						Survival growth (%)			Rosette diameter(cm)				Chlorosis (%)		
S/N	Line	0mM	50mM	100mM	150mM	50mM	100mM	150mM	0mM	50mM	100mM	150mM	50mM	100mM	150 mM(Description)
**1**	**WT**	32.8±0.5	27.5±0.2	17.3±0.8	0	78.57	49.42	0	11.2±0.2	9.2±0.3	7.2±0.5	0	50	95	–
**2**	**L1**	32.7±0.3	29.8±0.3	24.6±0.6	10.2±0.6	85.14	70.28	29.14	11.5±0.3	9.9±0.3	8.4±0.4	2.1±0.5	25	75	Bleachedpatches
**3**	**L2**	32.5±0.8	29.2±0.4	24.1±0.4	9.3±0.5	83.42	68.25	26.57	11.3±0.4	9.8±0.4	8.0±0.4	1.9±0.4	25	75	Bleached
**4**	**L3**	31.7±0.6	27.3±0.1	21.2±0.5	6.5±0.4	78.0	60.57	18.55	10.7±0.2	9.3±0.5	7.6±0.2	1.4±0.3	50	85	Maxbleached

Comparison of various growth parameters of WT (untransformed) and *WsSGTL1* transgenic *Arabidopsis* plants grown under normal (0 mM), 50 mM, 100 mM and 150 mM NaCl conditions. Salinity stress was imposed on 4-weeks-old soil-grown plants, which were irrigated with the indicated NaCl solutions every second day for 14 d. Data represents the mean and standard error (**±**SE) (*n = *35). Rosette diameter was calculated by comparing data from normal and salt treated plants. Similar results were obtained from three independent experiments. Extent of leaf chlorosis was visually rated on a scale percentage. Normal condition showed no chlorosis (data not presented). ‘0’ denotes no survival in case of WT.

### Measurement of Oxidative Stress - Comparison of SOD Activity and Relative Electrolytic Conductivity (REC) in Transgenic and WT Plants

**Figure 5:**
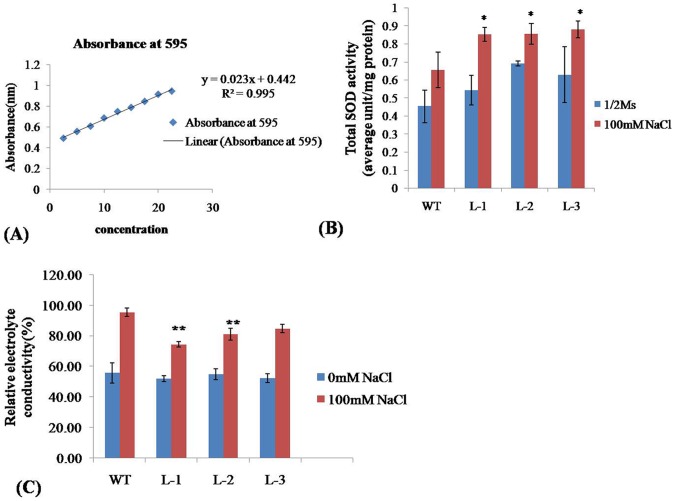
SOD activity and measurement of relative electrical conductivity. Standard calibration curve for SOD at 595 nm **(A).** SOD activity analysis in *WsSGTL1* transgenic lines of *A. thaliana* and WT (Col-0) plants. Values are expressed as mean (n = 3); errors bars show the SD for each experiment; (*) *P≤0.05* compared to WT (t-test) (**B**)**.** REC of *WsSGTL1* transgenic lines of *A. thaliana* and WT (Col-0). Values are expressed as mean (n = 3); errors bars show SD.

The transgenic lines expressing the WsSGTL1 protein remained green when exposed to higher NaCl concentrations in comparison to WT plants. The visual observations were confirmed by measuring SOD activity under normal conditions and after three weeks of growth on 100 mM NaCl. Under normal conditions, the level of SOD was higher in transgenic plants as compared to WT plants. Under salt stress, the level of SOD increased further as compared to WT plants (Figures 5 A, B; Table 2). The percentage increase in the levels of SOD in transgenic lines as compared to WT was: 30.7%, 32.30% and 35.38% for L1, L2 and L3 lines, respectively (Table 2). The results showed that under salt stress, the SOD activity of transgenic plants was much higher than the WT plants. A comparison of REC between transgenic and WT plants showed that the WT plants had a higher REC than the transgenic lines after salt stress (Figure 5C). The transgenic lines L1 and L2 showed significantly (P≤0.01) lower REC as compared to WT plants after salt stress. Higher SOD activity and lower relative electrolytic conductivity (REC) reflected that transgenic lines were adapted to salt stress.

**Table 2:** SOD activity in WT and transgenic lines of *A. thaliana*.

Genotypes	(½ MS )	(½ MS +100 mM NaCl)	Increase in SOD activityin100 mM NaCl	Increase in SODactivity (%)
WT	0.45±.09	0.65±.09	Reference	Reference
L1	0.54±.08	0.85±.03	0.85−0.65 = 0.2	30.7*
L2	0.69±.01	0.86±.05	0.86−0.65 = 0.21	32.30*
L3	0.62±.15	0.88±.04	0.88−0.65 = 0.23	35.38*

* SOD enzyme (units/mg protein) activities in transgenic and WT *Arabidopsis* under normal and salt (100 mM NaCl) stress conditions. Increase in SOD activity was compared with WT. Values are expressed as mean (n = 3); errors bars show the ±SD for each experiment. (*)*P<0.05* compared to WT (t test).

### Effect of Heat Stress on WT and Transgenic *A. thaliana*

**Figure 6:**
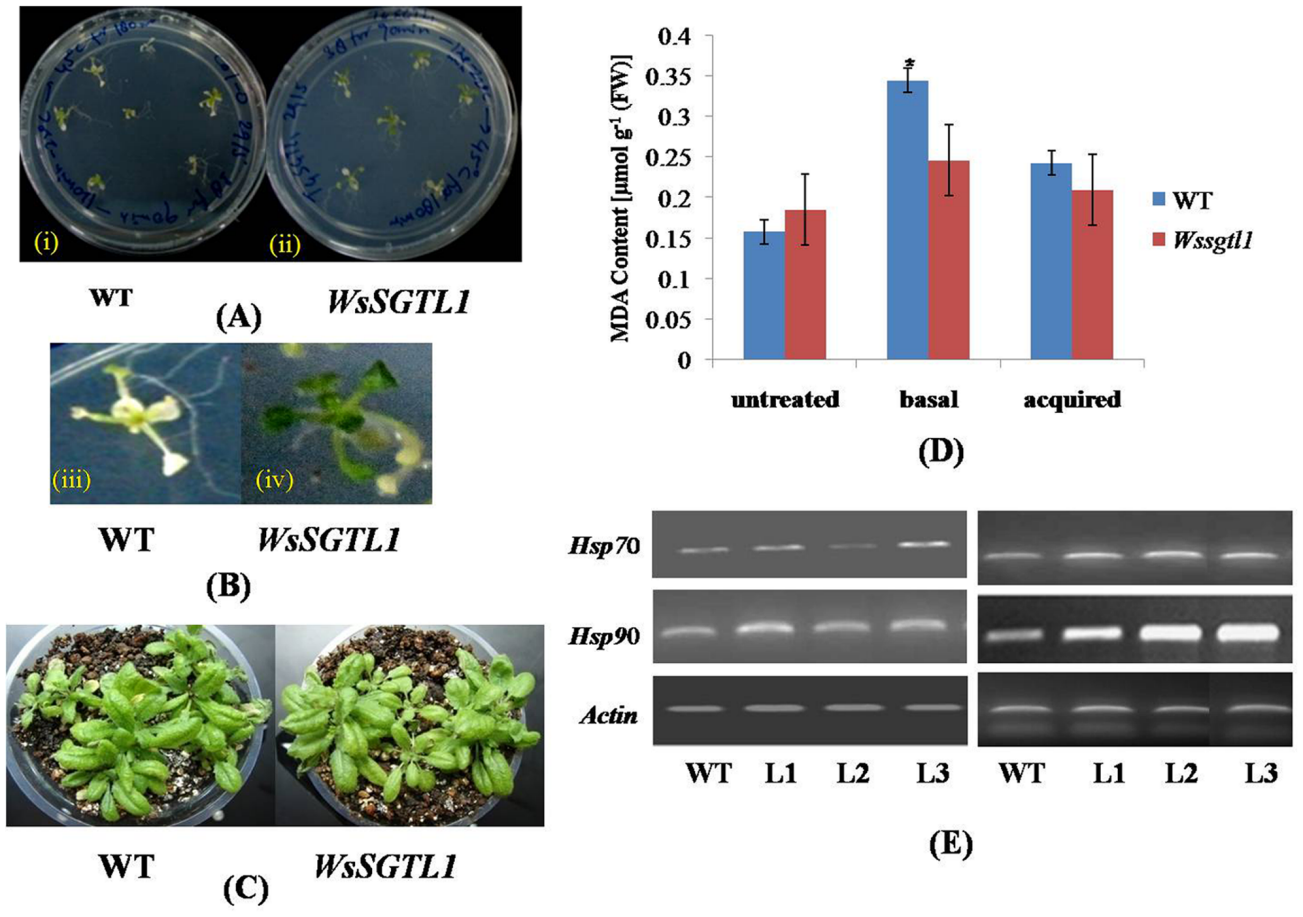
Phenotypes and heat tolerance of the transgenic plants. Thermotolerant phenotypes of WT and overexpression lines of *A.thaliana,* showing heat sensitivity at 7-d-old seedlings stage. Seedlings were grown on agar plates in light for 7 d and heated at 38°C for 90 min, cooled at room temperature for 120 min, and again heated at 42°C for 180 min (acquired thermotolerance). Percentage of survival of plants in relation to WT control plants on the same plate was determined 5 d after heat stress (**A–B**). Three-weeks-old soil grown plants exposed directly to 42°C for 60 min (basal thermotolerance). Photograph was taken after 7 day survival of the plants (**C**). Heat-induced oxidative damage in WT as compared to overexpression lines with decreased thermotolerance. Plants were heat treated as described in Figure (**A)**, and after 2 days of recovery, seedlings were harvested and stored in liquid nitrogen until the assay was performed. The MDA level determined from the overexpression lines of *A.thaliana* in relation to WT control on each plate was determined. Values are expressed as mean (n = 3); errors bars show the SD for each experiment. (*) *P≤0.05* compared to WT (t-test) (**D**)**.** Two-weeks-old seedlings of WT and all transgenic lines were used for RNA extraction. For heat stress, seedlings were kept at 42°C for 4 h before RNA isolation. The transcript level of two stress genes was determined by RT-PCR analyses. The stress genes used for the tests were *Hsp70, Hsp90* (**E**).

**Figure 7:**
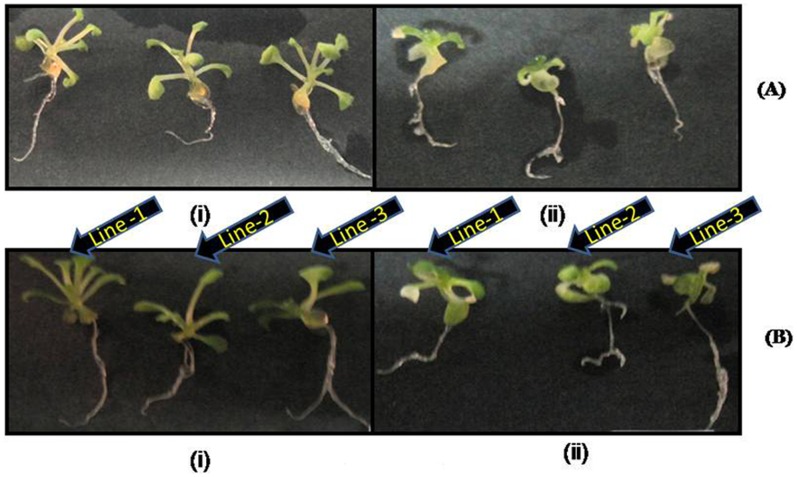
Phenotypes of 14-d-old seedlings of transgenic plants showing heat tolerance. Thermotolerant phenotypes of WT and overexpression lines of *A.thaliana,* showing heat sensitivity at 14-d-old seedling stage. Seedlings were grown on agar plates in light for 14 d and heated to 45°C for 180 min (basal thermotolerance). Photograph was taken after 7 day survival of plants before and after heat stress. (A) WT plants. (B) Transgenic plants.

Phenotypic analysis of WT and overexpression lines of *A. thaliana* showed heat sensitivity at 7-d-old seedling stage (Figure 6 A, B). Seedlings grown on agar plates in light for 7 d were heated to 38°C for 90 min, cooled at room temperature for 120 min, and then heated to 42°C for 180 min (acquired thermotolerance). We found 83.57% survival of transgenic plants in comparison to WT plants (Table 3) after heat stress. In another experiment, 14-days-old seedlings were exposed directly to 42°C for 180 min (basal thermotolerance), percentage of survival of transgenic plants was almost similar as above (Figure 7). Three-weeks-old soil grown plants, when exposed to 42°C for 180 min and after 7 days survived plants showed maximum number of bleached leaves in WT, whereas in transgenic lines (L1 and L2), very few leaves were bleached ((Figure 6C; Table 3). As a test of heat sensitivity and measure of oxidative damage, WT and transgenic lines of *A. thaliana* were assayed for accumulation of MDA. The results showed that transgenic lines had lower MDA content than WT, whether exposed to either of the heat stresses - acquired or basal (Figure 6D). The expression of *HSP70* and *HSP90* through semi quantitative PCR was also significantly increased in all lines upto 4 h of heat stress (Figure 6E).

**Table 3:** Survival of plants as determined under heat stress and cold stress in the plate based early analysis platform.

Heat stress					Cold stress			
S/N	Genotypes	7-day-old seedlings exposed to 38°Cfor 90 min, cooled at roomtemperature for 120 min. Againheated at 42°C for 180 min.Survival of plants after 5 days.		3-weeks-old plants exposedat 42°C for 180 min. Survivalof plants after 7 days.	14-days-old seedlingsat 4°C for 24 h (NA).Survival of plantsafter 7 days.		14-days-old seedlings at−1°C for 24 h (CA).Survival of plantsafter 2 days.	
		Average	Survival (% )	Description	Average	Survival (% )	Average	Survival (% )
**1**	**WT**	69.2±0.5	72.84	Bleached	93.2±0.2	98.1	73.2±0.7	77.05
**2**	**L1**	79.4±0.3	83.57	Green	94.5±0.7	99.47	82.5±0.3	86.84
**3**	**L2**	77.9±0.4	82	Green	93±0.5	97.89	83.1±0.8	87.74
**4**	**L3**	72.8±0.9	76.63	Green with bleached patches	91±0.4	95.78	79.3±0.2	83.47

Comparison of number of plants (WT and transgenic lines), surviving on exposure to heat stress (acquired thermo tolerance and basal thermo tolerance assay) and freezing stress (4°C and −1°C). Each experiment was repeated three times. 90 plants were tested in each experiment. Data represents the mean and standard error (±SE).

### Effect of Cold Stress on WT and Transgenic Lines

**Figure 8:**
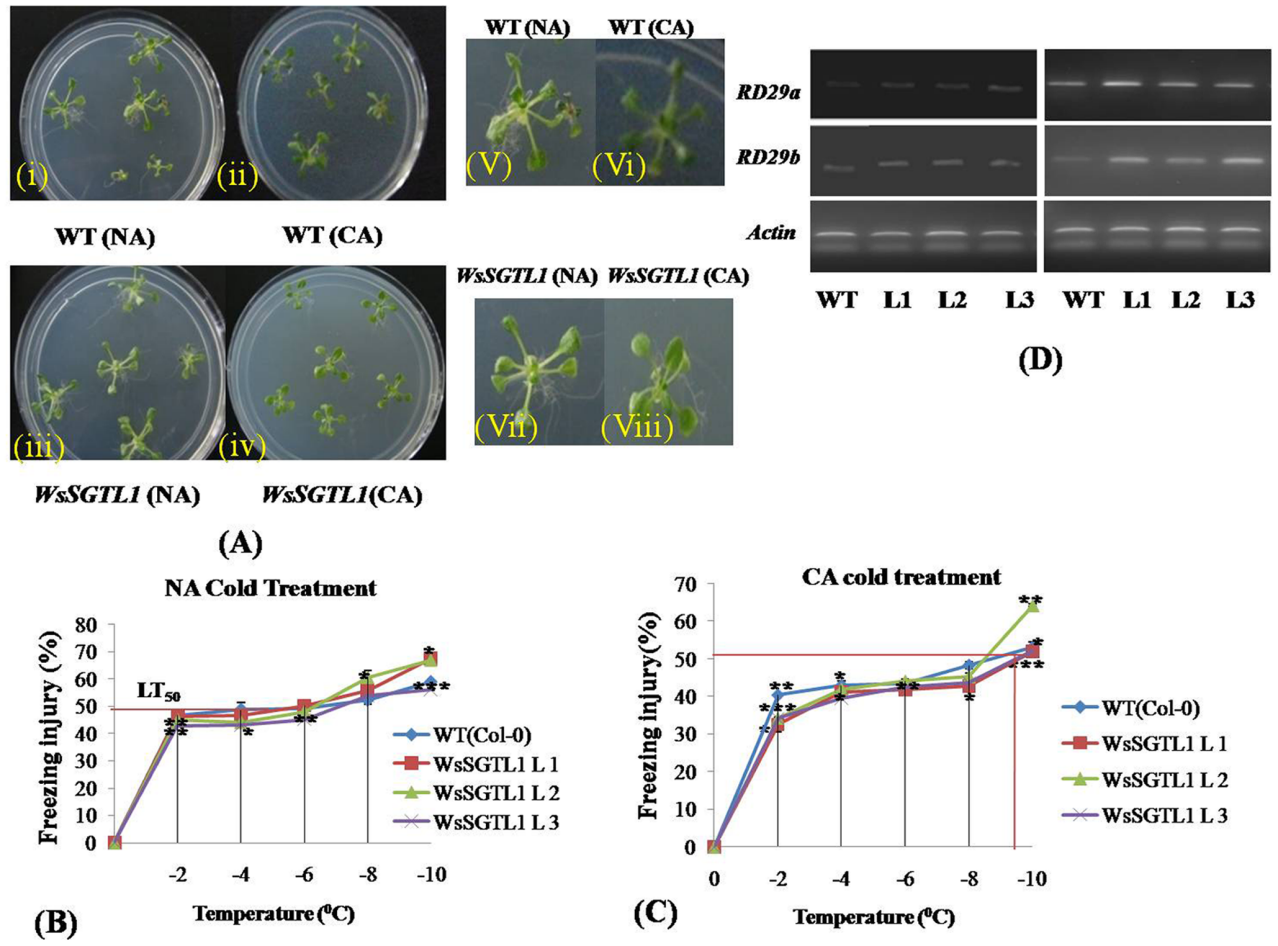
Phenotypes and cold tolerance of the transgenic plants. Cold stress was imposed on after 10–12 days of growth. Plates can be transferred directly to a freezing chamber set at −1±0.1°C in the dark to check the basal cold tolerance (NA). For cold acclimated (CA), the plates were first transferred to a cold room set at 4±2°C, under constant light for 7 days. Phenotypes of plants after 2 days score the survival of seedlings visually (**A**)**.** Freezing tolerance (*LT50*) of 7-days-old WT and transgenic NA seedlings (**B**) and CA seedlings (**C**). Values are expressed as mean (n = 3); error bars show the SD for each experiment; (*) *P≤0.05* compared to WT (t-test). Two-weeks-old seedlings of WT and transgenic lines were used for RNA extraction. For cold stress, seedlings were treated at 4°C for 24 h before RNA isolation. The transcript level of two stress genes was determined by RT-PCR analyses. The stress genes used for the tests were *RD29a, RD29b* (**D**)**.**

Phenotypic analysis for basal cold tolerance (NA, non acclimated), was performed in growing plates of 12-days-old seedlings of WT and overexpression lines of *A.thaliana* which were transferred directly to a freezing chamber at −1°C. For cold-acclimated (CA), the plates were first transferred to a cold room set at 4±2°C, under constant light for 7 days (Figure 8A). Under NA condition, survival of transgenic plants increased significantly as compared to WT, whereas, in case of CA condition, survival percentage of transgenic plants was more than WT but it was not significant (Table 3). We explored the ability of the plants to acclimatize to cold conditions by measuring ion leakage and survival rates after one week of acclimation at 4°C. The freezing tolerance LT_50_ (temperature of 50% electrolytic leakage) of NA transgenic and WT seedlings was about −6°C (Figure 8B). Both the genotypes exhibited increase in LT_50_ during a CA treatment (Figure 8 C). After 7 days of CA, the LT_50_ of transformed plants was 1°C higher (more negative) than that of the WT plants. Moreover, the relative level of freeze-thaw injury of transgenic plants at various sub-freezing temperatures (−2 and −8°C) was lower than that of WT plants and more significant (*P≤0.01*). We assessed the expression of *RD29A* and *RD29B* which are cold responsive genes and under the control of *DREB/CBF* elements (transcriptional activator of *cor* genes). When determined through semi quantitative PCR, the *RD* genes showed increase in expression in transgenic lines under cold condition (Figure 8 D).

### Differential Effects of Salt, Heat and Cold Treatments on Chlorophyll Fluorescence in Both WT and Transgenic Plants

**Figure 9:**
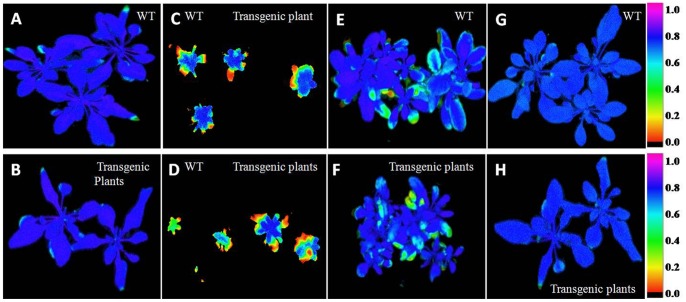
Chlorophyll Imaging Fluorescence measurements. Fv/Fm values in WT and *WsSGTL1*transgenic lines of *A.thaliana* after normal growth condition of WT (**A**)**.** Normal growth condition of transgenic line of *WsSGTL1* (**B**)**.** Salt treatments of WT and transgenic lines of 50 mM (**C**)**.** Salt treatments of WT and Transgenic lines of 100 mM NaCl (**D**)**.** Heat treatment of (42°C) in WT (**E**)**.** Heat treatment of (42°C) in transgenic lines of *A. thaliana* (**F**)**.** Cold treatment (4°C) in WT (**G**)**.** Cold treatment of (4°C) in transgenic lines of *A. thaliana* (**H**)**.** Salt treatment started after shifting the 14 days seedling plants into the pot for three weeks and all image analysis was done after three week potted plants.

The chlorophyll fluorescence imaging has been shown under the selected fluorescence parameters (Figure 9), and the variability of the fluorescence response parameters has been shown in (Figure S9) through spider plots. Data has been presented in Table S2. The three spider plots show the dynamics of conventional fluorescence parameters during the abiotic stress of *A. thaliana* by salt, heat and cold (Figure S9 A-C). Each spider plot consisted of different parameters, some measured with dark-adapted plants (Fv/Fm) while others with low light intensity (80 µmol m^−2^ s^−1^). The distance from the centre of the spider plot indicates the relative change of the fluorescence parameter during the treatment. The changes were measured by comparing the signals in the untreated plants with the treated plants. Clearly, under the normal conditions both genotypes showed approximately similar fluorescence response in spider plots (Figure S9C). In spider plot B which represents transgenic plants, the florescence dynamics of 100 mM NaCl was different from the control, while in all other stress conditions, the dynamics did not change much (Figure S9B). In spider plot A, which represented WT plants, the florescence dynamics changed with heat, cold or salt stresses as compared to the control conditions (Figure S9A). In other words, the transgenic plants showed adaptation to stress conditions and the dynamics did not change much during stress. Level of significance considered as *P≤0.05.*

### Biochemical Analysis of Transgenic and WT Plants Through HPLC and Enzyme Assay

**Figure 10:**
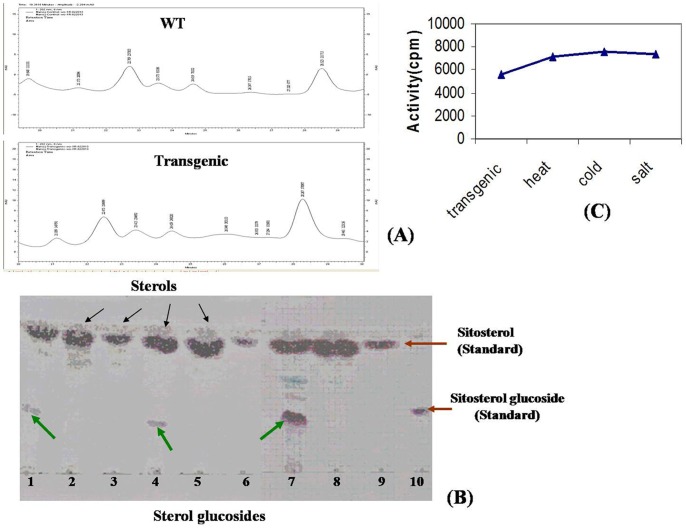
Analysis of sterols in WT and *WsSGTL1* transgenic *Arabidopsis* lines by HPLC, TLC and enzyme assay. Comparative analysis of sterols in transgenic *Arabidopsis* lines expressing *WsSGTL1* under the control of the cauliflower mosaic virus 35S promoter. Contents of campestrol, stigmasterol and sitosterol in the leaves of WT and different transgenic lines (**A**)**.** HPLC purified plant extracts spotted on TLC plate (Lane 1–10) Lane 1 & 4: WsSGTL1 Transgenic plants; Lane 7: Transgenic plant after salt stress (100 mM for 24 h); Lane 2,3,5,6,8: Wild type plants; Lane 9 & 10: Standards Black arrows indicate sterols whereas green arrows indicate sterol glycosides; red arrows indicate standards (**B**)**.** The crude enzyme activity was determined as cpm or counts per minute and expressed as mCi mmol-1. It was observed that in transgenic plants the enzyme activity was about 5500 mCi mmol-1 which increased to about 7500 mCi mmol-1 in stress conditions as done by radiolabelled enzyme assay (**C**).

The sterol peak separated by HPLC (Figure 10A; Figure S10) showed two types of bands in TLC. The upper band was of sterol while the lower of glycosylated sterol. β-sitosterol glucoside was used as standard for the glycosylated products formed (Figure 10B) and sitosterol was used as non-glycosylated standard. In wild type plants only one band was seen which correspond to sterols and in transgenic plants two bands were seen which correspond to sterol and glycosylated sterol. The R_f_ values of the run samples were between 0.4 and 0.6. As the enzyme could not be purified for specific activity, we could only check the enzyme activity in crude extacts by scintillation counter expressed as mCi mmol^−1^. As compared to WT, the enzyme activity was more in the transgenic plants which increased from approx 5500 mCi mmol^−1^ to 7500 mCi mmol^−1^ in stress conditions (Figure 10C).

### Expression of *WsSGTL1* in Transgenic Plants

The expression of *WsSGTL1* in transgenic plants under different abiotic stress like heat stress (42°C), cold stress (4°C) and different concentration of NaCl 50,100 or 150 mM was detected by real-time PCR. No *WsSGTL1* expression was detected in WT plants. Under different stress conditions all the transgenic lines showed 1.5 to 2 fold increase in expression in response to heat, cold and salt stress as compared to non stressed condition (Figure S11).

### Promoter Sequence Analysis

Since the gene was responsive to abiotic stress, we tried to find out the presence of stress responsive elements in *SGT* genes. The sequence (Figure S4B) analysis of promoter showed that it contained ABRE like sequence form (−199 to −195) required for etiolation-induced expression of *ERD1* (early responsive to dehydration 1) in *Arabidopsis*. It also contained the CCAAT box found in the promoters of heat shock proteins. The GT-1 motif GAAAAA was also found in the promoter which is responsible for salt induced *SCaM-4* gene expression (Table 4). Hence the promoter analysis showed that it contained cis-acting elements which are responsible for abiotic stress response.

**Table 4:** Analysis of different cis-acting regulatory elements in the promoter of *WsSGTL1* using PLACE software.

Factor or Site Name	Location(Strand)	SignalSequence	Description
ABRELATERD1	site 1212 (+)	ACGTG	ABRE-like sequences (from −199 to −195) required for etiolation - induced expression of erd1(early responsive to dehydration) in *Arabidopsis*
ARFAT	site 801 (+)	TGTCTC	ARF (auxin response factor) binding sites found in the promoters of primary/early auxin response genes of *A. thaliana*. This element was enriched in the 5′ flanking region of genes upregulated by both IAA and BL.
CCAATBOX	site 661 (+)	CCAAT	Common sequence found in the 5′-non-coding regions of eukaryotic genes; “CCAAT box” found in the promoter of heat shock protein genes; located immediately upstream from the most distal HSE of the Promoter, “CCAAT box” act cooperatively with HSEs to increase and the hs promoter activity.
ELRECOREPCRP1	site 37 (−)	TTGACC	EIRE (Elicitor Responsive Element) core of parsley(P.c) PR1 genes; consensus sequence of elements W1 and W2 of parsley PR 1-1 and PR 1–2 promoters; Box W1 and W2 are the binding site of WRKY1 and WRKY2 respectively; ERE; “WA box”; one of the W boxes found DE in the Parsley (P.c) WRKY1 gene promoter; Required for elicitor responsiveness.
GT1GMSCAM4	site 78 (−)	GAAAAA	“GT-1 motif” found in the promoter of soybean (Glycine max) CAM isoform,SCAM -4; plays arole in pathogen and salt induced Scam-4 gene expression
WBOXATNPR1	site 144 (+)	TTGAC	“W-box” found in promoter of *A. thaliana* NPR1 gene; Located between +70 and +79 in tandem; They were recognized specially by Salycilic acid (SA)-induced WRKY DNA binding proteins.

### Bioinformatics Analysis

The 3D structures as built up on Phyre 2 online server using ‘c3hbjA’ backbone showed that both *WSSGTL1* and *AtSGT* contained a sterol binding domain AIIANPPAY and a UDP-sugar binding domain VVHHGGAG (Figures 2A,B). The structures were superimposed as the variability was 1.423 Å (Figure 2C, Figure S5C) which showed their functional similarity. The full-length *WsSGTL1* cDNA sequence (GeneBank NO. DQ356887) comprised 2532 bp, with an ORF of 2103 bp that encodes 701 amino acids followed by stop codon, a 337-bp 5′untranslated region (UTR), and a 89-bp 3′ UTR containing a predicted AATAAA polyadenylation signal. The basic properties of *WsSGTL1*, as predicted by Protparam, are 17.4 k Da MW, a pI of 4.94 and a GRAVY value of 0.682, which indicated a hydrophobic monomer protein (Figure S12). Conserved PSBD and UGT prosite motifs were present in the deduced amino acid sequence of *WsSGTL1.* Two putative transmembrane domains (from 369 to 387 and 406 to 432 amino acid residues) were detected in *WsSGTL1* by **tmap (** http://www.bioweb.pasteur.fr/seqanal/interfaces/tmap.html **)**. The presence of putative transmembrane domains suggested the association of *SGTL1* with membranes [15].

## Discussion

Plants have evolved an extraordinary capacity to perceive changes in their external environment and adapt rapidly to maximize opportunity and minimize risk [29]. Previously, in a review on *SGT*s, we have reported that sterols are important components of cell membranes, hormones and secondary metabolites which play important roles in defense mechanism of plants [12]. It is well documented that sterol glycosyltransferases (SGTs) are enzymes involved in sterol modifications and play an important role in metabolic plasticity during adaptive responses. This plasticity depends on the integration of growth, development, and metabolism, and the evolution of diverse mechanisms to regulate cellular homeostasis. These modifications help in adaption of the plants to fit better in the environment. In the publications reported from our lab, the *WsSGTL1* gene of *W.somnifera* has been shown to be involved in different biotic and abiotic stresses [15], [1]. The present study on *WsSGTL1* gene expression analysis was mainly focused on responses to abiotic stresses.

After transformation with *WsSGTL1*, we observed that the transgenic *Arabidopsis* plants were more tolerant to salt stress than WT, as evident from their growth parameters like germination, root length and shoot weight. During salt stress, major lipid changes are induced in the plasma membrane fraction and a change in the fatty acid composition resulting in a concomitant increase in the degree of saturation. This change has profound implications for membrane functioning under salt stress. Sterol glycosyltransferases help to maintain sterol: glycosylated, sterol: acylated glycosterol ratio of membrane sterols and are helpful in protecting membrane physiology. It is generally accepted that the first deteriorating change during stress injury is an alteration in the structure and function of cell membranes. The responses of plants to salt stress are complex and involve changes in their morphology, physiology and metabolism. In many plants, alterations in lipids, particularly in phospholipids and sterols were observed, as a result of water stress [30] or salt treatment [31]. The most common and widely reported stress-regulated genes are *LEAs* or *LEA*-like genes that encode late embryogenesis abundant proteins [32] and salt overly sensitive genes *(SOS3*) [33]. Semi quantitative PCR analysis showed that *LEA4-5* and *SOS3* genes are highly expressed (Figure 4C) during salt stress.

During salt stress, the production of significant amount of reactive oxygen species (ROS) causes damage to proteins, lipids, nucleic acids and other sites of cells. This process is a lethal factor for salt sensitive plants. Tolerant plants have evolved an antioxidant defense system like superoxide dismutase (SOD) which protects them against oxidative damage. Therefore, antioxidant activity, especially SOD (which are scavengers of ROS), can be used as a biochemical marker to evaluate oxidative stress during salt tolerance. Gill et al. [34] overexpressed *PaSOD* from *Potentilla* in *Arabidopsis* and showed that it improves salt tolerance during germination and growth. Under normal conditions, the level of transgene (*PaSOD*) is higher than WT plants. However, in the presence of NaCl stress, the levels are much higher in transgenic plants as compared to the WT plants. In agreement to this the SOD activity increased 1.19 to 2 folds in transgenic lines as compared to WT plants under salt stress in our study. Under normal conditions also, i.e. in the absence of salt stress, the level of SOD was more in transgenic plants. It is inferred that the transgene *WsSGTL1* triggers the antioxidant defense machinery much before the onset of oxidative stress by signaling; resultantly the SOD levels are higher in the transgenics. At the onset of salt stress, the antioxidant response is elevated which further increased SOD levels. This kind of protective mechanism appears to do limited damage to cell components in transgenic plants as compared to WT plants. In a study by Napoli et al. [35], glycosylation enhances the SOD activity to prevent lipid peroxidation in case of human low density protein. Similarly glycosylation of sterols such as brassinosteroids takes place *in planta* which is regulatory defense mechanism controlling homeostasis against any biotic or abiotic stimuli [36]. After salt stress, there was higher relative weight gain in the transgenic plants as compared to the WT and significantly lower REC, which may be due to higher stability of cell membrane in the transgenics. It is speculated that the overexpression of *WsSGTL1* is associated with the stability of cell membranes. In another study reported by Lin et al. [37], it is demonstrated that two glycosyltransferase genes *TaUGT1* and *TaUGT2* are upregulated under high NaCl stress. Our results showed that *WsSGTL1*, like other GT family proteins is involved in resistance to abiotic stresses and it improved the tolerance of transgenic *Arabidopsis* to salt stress.

MDA is an end product of lipid peroxidation in biomembranes and the MDA content usually reflects the level of lipid peroxidation that indirectly reflects the extent of membrane injury. High level of MDA correlates with high level of oxidative damage to lipid membranes [38]. Our study showed that *WsSGTL1* transformed lines had significantly lower MDA contents than the WT plants upon being exposed to heat stress. Phenotypic analysis of *A. thaliana* under heat stress showed that transgenic lines were more tolerant than WT plants, as the recovery after the stress was not only faster in transgenic lines but plants grew healthier than the WT plants. It may be hypothesized that overexpression of *WsSGTL1* enhances heat tolerance in the transgenic *Arabidopsis* plants. *A. thaliana* thermosensitive mutant *atts02* reveals an important role of galactolipids in thermotolerance [39]. It is well known that transcriptional induction of heat shock genes is mediated by the heat shock transcription factors (HSFs) in eukaryotes [40] The HSFs are activated by multimerisation and phosphorylation, bind to the promoters of heat shock genes and stimulate their transcription. Earlier reports from *Myxoamoeba* and human fibroblastoma cell lines showed the activation of *SGTs* and the production of sterol glucoside following heat stress [41], [42], [43]. The activation of sterol glycosyltransferase and the production of sterol glycosides are important mechanisms in signal transduction pathway. They lead to the synthesis of heat shock proteins during heat stress [43], [12] suggesting the role of *SGT* genes during heat stress. In agreement to these studies, we observed the elevated expression of the heat shock protein HSP70 and HSP90 under heat stress. This suggests that the overexpression of *WsSGTL1* is associated with heat tolerance in transgenic *Arabidopsis*.

Membrane sterols play a very important role in cold stress. Our previous study showed increase in the expression of *WsSGTL*s during cold stress, which may regulate the level of sterols and their glucosides during cold stress, in the leaves of *W. somnifera* [1]. Our present study on *WsSGTL1* transgenic *Arabidopsis* supported this observation by finding elevated transcript level of *WsSGTL1* within 24 h in cold stress and during freezing tolerance test. Plant tissues freeze extracellularly as a result of a heterogeneous ice nucleation events [44]. Since, the water potential of ice is less than that of unfrozen water, cellular water moves down the gradient to extracellular spaces resulting in cellular dehydration. Plant cell membrane systems are the predominant site of freezing injury. Such damage is associated primarily with severe dehydration. Thus, a key function of CA (cold acclimation) is to stabilize those membranes via multiple mechanisms. For example, alterations in membrane lipid composition are correlated with membrane cryostability [45]. It is reported that the concentration of membrane sterols increases during cold acclimation in tolerant rye cultivars [46]. In the present study, no difference was observed in the ‘constitutive’ freezing tolerance (LT_50_) of *WsSGTL1* transgenic and WT *Arabidopsis* plants (Figure 8B, C). After 8 days of CA, however, the WT plants were consistently and significantly less freeze-tolerant than the *WsSGTL1* transgenic plants based on both the cold acclimated LT_50_ (1°C lower) as well as higher injury conditions (at −2 to −8°C freeze-thaw stress). Similar data was obtained from three separate experiments. Furthermore, *WsSGTL1* transgenic plants were more freeze tolerant throughout the CA treatment, as is evident from the time-course data. Several enzymes involved in sterol metabolism, like, wheat ESTs have been over expressed, suggesting major lipid modifications in membranes during CA [47]. Due to the role of sterols in membrane fluidity and permeability and also the phospholipid dependence of UDP Glc:sterol glucosyltransferase, SGTs are considered to have a positive role in adapting plants to temperature stress [48]–[51]. Because sterol content of the plasma membrane changes in response to environmental conditions, alterations in the sterol compositions of plasma membranes may play a role in CA process [52]. This is supported by the fact that *SGT*s of *A.thaliana*, e.g. *UGT80B1* transcript gets slightly upregulated during cold stress, as shown by gene expression data from micro-array experiments [53]. Hence, *SGT*s, which bring about the modification of sterols, play an important role in coping up with heat and cold stress. These results suggested that WT plants had lower CA ability than the transformed *Arabidopsis* plants.

Chlorophyll fluorescence and analyses of PSII (Fv/Fm) are useful for the monitoring of salt, heat and cold stress. In a previous report, the physiological changes have been measured by photoinhibition at high salinity [54]. This concept was strongly supported by our results. When plants were exposed to salt-stress, their reaction centers were damaged (photochemically inactive), thus reducing electron transport capacity in PSII. Similar results were previously reported in *Jatropha curcas* [55] and barley [56]. In our study, both light and dark reactions of photosynthesis were impaired much more in WT than in *WsSGTL1 A.thaliana* since the performance indices of photochemical Y (NPQ) reactions were much lower in stress-exposed plants than in control plants (Figure S13).

High temperature stress reduced the Fv/Fm ratio, as well as Y (II) indicating a structural and functional disorder of the photosynthetic apparatus and damage to the PSII. In our study WT plants showed lowering of Fv/Fm as compared to transgenic plants. Similar findings were observed in tall fescue [57] and pea leaves [58]. Reductions in the Fv/Fm ratio and Y (II) under high temperature stress suggest that an important portion of the PSII reaction centre was damaged in the WT. These damages are associated with structural modifications in PSII, especially in D1 protein, which in conditions of heat stress is phosphorylated and degraded afterwards [59]. Transgenic *WsSGTL1* had higher Fv/Fm ratios and Y (II), during high temperature stress than WT, indicating that the photosynthetic apparatus in WT was more susceptible to heat stress than in *WsSGTL1*. In cold stress there was no major difference between PSII (measured as Fv/Fm) ratio after 2 days of recovery in both WT and transgenic *WsSGTL1* plants. The spider plots representing fluorescence dynamics showed that in transgenic plants the dynamics does not change much during stress conditions as compared to the WT plants indicating that the transgene confers tolerance to stress conditions (Figure S9). To confirm role of *WsSGTL1* in abiotic stress we performed radiolabelled enzyme assay in transgenic plants, which showed mild increase in enzyme activity under stress conditions (Figure 10 C). HPLC-TLC indicated the formation of sterol glucosides in transgenic plants and in stress conditions (Figure 10 B).

Since, the transgene *WsSGTL1* was involved in abiotic stress tolerance in *Arabidopsis,* we aimed to clone the promoter of this gene from *W. somnifera* and see whether stress responsive elements are present in the promoter. On sequencing we found that the promoter contains abiotic stress responsive elements which confirmed our hypothesis that *WsSGTL1* is involved in stress. Furthermore, we attempted to elucidate the function of the gene by using bioinformatic approach and we found that the 3D protein models developed by online servers (Phyre 2) contained two domains i.e. one sterol binding domain and another UDP sugar binding domain. We superimposed the 3D models of *WsSGTL1* with *AtSGT* which is an *Arabidopsis* glycosyltransferase and we found that these two were superimposable with only 1.42 A° difference. This confirmed that *WsSGTL1* is a glycosyltransferase with functional similarity to *AtSGT*.

The present work has provided novel information enhancing the knowledge about the involvement of *WsSGTL1* gene of *W. somnifera* in response to environmental stresses. The complexity in the proposed function of *WsSGTL1* and the limited information on the biological roles of plant *SGTs* are challenging aspects for further investigation. Our analyses of the transgenic plants were focused on the role of this gene in response to different abiotic stresses and identification of promoter for *SGTL1* gene of *W. somnifera.* Presence of salt responsive, heat shock and cold inducible elements in the *WsSGTL1* promoter region and chlorophyll image analysis postulates that these genes are responsible for stress tolerance in *W. somnifera*. The presence of sterol glucosides in transgenics, enzyme activity and stress modulation of *WsSGTL1* gene expression suggests its specific functional recruitment under environmental challenges.

## Supporting Information

Figure S1 **Schematic diagram showing T-DNA region of binary vector pBI121 used for transformation.** The *WsSGTL1* gene was inserted at the XbaI-SacI site in sense orientation. Promoter DE*CaMV35S* (600 bp), *WsSGTL1* (2.1 Kb) and the restriction sites were assembled and transformed in *A. thaliana.*
(TIF)

Figure S2 **Colony PCR:** Analysis of *WsGTL*1 by *WsSGTL*1 (F) and *WsSGTL*1(R) primers; C1 negative control (lane 1), C2, C3, C4 and C5 positive colony (lane 2–5).
(TIF)

Figure S3 **Selection of transgenic** ***WsSGTL1*** **overexpression lines of** ***A.thaliana.*** **(A)** T_1_ generation transgenics in ½ MS media supplemented with 50 µg ml^−1^ kanamycin. **(B)** PCR analysis of 12 positive transgenic lines by Gene Specific Primers in T_2_ generation (amplicon size 2.1 Kb).
(TIF)

Figure S4 **A Genome walking PCR with adaptor primers and gene specific primers.** PCR products from non template control (lane 1), nested control (lane 2), AP1+GSP1 (lane 3), AP2+GSP2 (lane 4), AP1+GSP3 (lane 5) and AP2+GSP4 (lane 6) are shown. M;λ *HindIII/EcoRI* double digested DNA molecular weight markers. **B. Promoter sequence of WsSGTL1.** Nucleotide sequence of *WsSGTL1* promoter including intronic region (shown as red color) and overlapping sequence of *SGTL1* (shown as green color).
(ZIP)

Figure S5 **Secondary structure and alignments** **(A)** Secondary structure of *WsSGTL1* ***.*** **(B)** Secondary structure of *AtSGT* ***.*** **(C)** Alignment of *WsSGTL1* and *AtSGT*.
(ZIP)

Figure S6 **Germination rate and early seedling development of both WT and overexpression lines of** ***A.thaliana*** **under salt stress.** **(A)** Percentage of germinating seeds of WT and *WsSGTL1* overexpression lines (L1, L2 and L3) of T3 plants grown on MS medium. **(B)** Supplemented with 50 mM NaCl. **(C)** With 100 mM NaCl. **(D)** With 150 mM NaCl. Data of germination recorded after 7 days of germination. Values are percentage germination ± SE, n = 60, (**) for *P≤0.01* (***) for *P≤0.001* or *0.005* significantly different from the control (t-test) from three independent experiments.
(TIF)

Figure S7 **Phenotypic differences between** ***WsSGTL1*** **overexpression lines of** ***A. thaliana*** **and WT (Col 0).** **(A)** Seedlings after 14 days of germination grown with 0 mM NaCl. **(B)** Seedlings grown after 7 days of germination (from L to R); with 50 mM NaCl; with 100 mM NaCl; with 150 mM NaCl.
(TIF)

Figure S8 **Data of phenotypic comparison of WT plants and** ***WsSGTL1*** **overexpression lines of** ***A. thaliana*** **after 14 days of germination.** Comparison of shoot length, root length, shoot weight and leaf count of WT and *WsSGTL1* overexpression lines of *A. thaliana* grown on MS medium supplemented with 50 mM NaCl, 100 mM NaCl and 150 mM NaCl. (A) Average shoot length.(B) Average root length. (C) Average shoot weight. (D) Average number of leaves. Values are mean ± SE, n = 10, (*) for *P≤0.05*, (**) for *P≤0.01,* (***) for *P≤0.001* or *0.005* significantly different from the control (t-test).
(TIF)

Figure S9 **Spider plots of chlorophyll fluorescence.** Spider plots showing relative changes of mean values of selected fluorescence parameters of maximum photosynthetic efficiency (Fv/Fm), photosynthetic yield Y(II), excitation pressure 1-Y(II), total nonphotochemical quenching (NPQ), regulated heat dissipation Y (NPQ), and unregulated heat dissipation Y (NO) under different abiotic stress of both the genotype of *A. thaliana.* **(A)** Wild type plants(Col-0)**.** **(B)** *WsSGTL1* transgenic lines**.** **(C)** Both WT and transgenic lines under normal growth conditions**.** Data from normal growth (control condition) were adjusted to 1.0, and all other parameters were calculated as fold-changes.
(TIF)

Figure S10 **HPLC chromatogram of standard sterol compounds.** HPLC chromatogram of standard sterol with retention time Campesterol: 23.477, Stigma sterol: 24.619, Sito sterol: 28.480
(TIF)

Figure S11 **Relative expression of** ***WsSGTL1*** **through real time PCR.** Relative expression of *WsSGTL1* in transgenic *A.thaliana* regulated by *CaMV35S* promoter under salt, heat and cold stress. Plants were treated with 0, 50, 100 or 150 mM NaCl for 24 h. *WsSGTL1* expression was not detected in WT plants grown under both with or without salt stress. While it was markedly increased in L1 and L2 lines and slightly increased in L3 line under salt stress. At 150 mM NaCl stress, expression level was low. The seedlings were exposed to 42°C for 4 h to provide heat stress, while at 4°C for 24 h for cold stress. The expression of L1 and L2 was remarkably increased as compared to non treated plants.
(TIF)

Figure S12 **Preliminary properties of the** ***WsSGTL1*** **protein predicted by ProtParam.**
(TIF)

Figure S13 **Photosynthetic yield [Y (II)] and total heat dissipation (NPQ) under different abiotic stress conditions. (A)** In WT and transgenic lines of *A.thaliana,* photosynthetic yield decreased at 50 and 100 mM NaCl but in WT its values decreased significantly due to stress (*P≤0.01)*. **(B)** Total heat dissipation in WT decreased significantly (*P≤0.01)* but not under cold stress, whereas transgenic lines were much affected during salt, heat and cold stress.
(TIF)

Table S1 **List of primers used in present study.**
(DOCX)

Table S2 **Effects of different abiotic stress on both genotypes of** ***A. thaliana*** **(WT and** ***WsSGTL1*** **transgenic lines) in reference to fluorescence parameters.** Maximum quantum yield of PSII photochemistry (Fv/Fm), effective quantum yield of PSII Y (II)), total heat dissipation (NPQ), yield of regulated heat dissipation Y (NPQ), and yield of unregulated heat dissipation Y(NO).
(DOCX)

## References

[1] Chaturvedi P, Mishra M, Akhtar N, Gupta P, Misra P, et al (2012) Sterol glycosyltransferases-identification of members of gene family and their role in stress in *Withania somnifera* . Mol Biol Rep 39: 9755–9764. 22744427 10.1007/s11033-012-1841-3

[2] Matsuda H, Murakami T, Kishi A, Yoshikawa M (2001) Structures of withanosides I, II, III, IV, V, VI, and VII, new withanolide glycosides, from the roots of Indian *Withania somnifera* DUNAL and inhibitory activity for tachyphylaxis to clonidine in isolated guinea-pig ileum. Bioorg Med Chem 9: 1499–1507. 11408168 10.1016/s0968-0896(01)00024-4

[3] Zhao J, Nakamura N, Hattori M, Kuboyama T, Tohda C, et al (2002) Withanolide derivatives from the roots of *Withania somnifera* and their neurite outgrowth activities. Chem Pharm Bull 50: 760–765. 12045329 10.1248/cpb.50.760

[4] Jayaprakasam B, Nair MG (2003) Cyclooxygenase-2 enzyme inhibitory withanolide from *Withania somnifera* leaves. Tetrahedron 59: 841–849.

[5] Ghosal S, Lal J, Radheyshyam S (1989) Immunomodulatory and CNS effect of sitonidosides IX and X, two new glycowithanolides from *Withania somnifera* . Phytother Res 3: 201–206.

[6] Bhattacharya SK, Bhattacharya A, Sairam K, Ghosal S (2000) Anxiolytic-antidepressant activity of *Withania somnifera* glycowithanolides: an experimental study. Phytomedicine 7: 463–469. 11194174 10.1016/S0944-7113(00)80030-6

[7] Mirjalili MH, Moyano E, Bonfill M, Cusido RM, Palazon J (2009) Steroidal lactones from *Withania somnifera*, an ancient plant for novel medicine. Molecules 14: 2373–2393. 19633611 10.3390/molecules14072373 PMC6255378

[8] Chen L-X, Hao He, Feng Qiu (2011) Natural withanolides: an overview. Nat Prod Rep 28: 705–740. 21344104 10.1039/c0np00045k

[9] Chen RJY, Chung T-y, Li F-y, Lin N-h, Tzen JTC (2009) Effect of sugar positions in ginsenosides and their inhibitory potency on Na+/K+-ATPase activity. Acta Pharmacol Sin 30(1): 61–69. 19060914 10.1038/aps.2008.6 PMC4006530

[10] Schumacher K, Chory J (2000) Brassinosteroid signal transduction: still casting the actors. Curr Opin Plant Biol 3: 79–84. 10679450 10.1016/s1369-5266(99)00038-2

[11] Poppenberger B, Fujioka S, Soeno K, George GL, Vaistij FE, et al (2005) The UGT73C5 of *Arabidopsis thaliana* glucosylates brassinosteroids. Proc Natl Acad Sci USA 102: 15253–15258. 16214889 10.1073/pnas.0504279102 PMC1257699

[12] Chaturvedi P, Misra P, Tuli R (2011) Sterol glycosyltransferases–the enzymes that modify sterols. Appl Biochem Biotechnol 165: 47–68. 21468635 10.1007/s12010-011-9232-0

[13] Bartwal A, Mall R, Lohani P, Guru SK, Arora S (2012) Role of secondary metabolites and brassinosteroids in plant defense against environmental stresses. J Plant Growth Regul DOI 10.1007/s00344–012–9272.

[14] Glombitza S, Dubuis PH, Thulke O, Welzl G, Bovet L, et al (2004) Crosstalk and differential response to abiotic and biotic stressors reflected at the transcriptional level of effector genes from secondary metabolism. Plant Mol Biol 54: 817–835. 15604654 10.1007/s11103-004-0274-3

[15] Sharma LK, Madina BR, Chaturvedi P, Sangwan RS, Tuli R (2007) Molecular cloning and characterization of one member of 3beta-hydroxy sterol glucosyltransferase gene family in *Withania somnifera* . Arch Biochem Biophys 460: 48–55. 17324374 10.1016/j.abb.2007.01.024

[16] Madina BR, Sharma LK, Chaturvedi P, Sangwan RS, Tuli R, et al (2007) Purification and physico-kinetic characterization of 3beta-hydroxy specific sterol glucosyltransferase from *Withania somnifera* (L) and its stress response. Biochim Biophys Acta 1774: 392–402. 17293176 10.1016/j.bbapap.2006.12.009

[17] Madina BR, Sharma LK, Chaturvedi P, Sangwan RS, Tuli R (2007) Purification and characterization of a novel glucosyltransferase specific to 27beta-hydroxy steroidal lactones from *Withania somnifera* and its role in stress responses. Biochim Biophys Acta 1774: 1199–1207. 17704015 10.1016/j.bbapap.2007.06.015

[18] Clough SJ, Bent AF (1998) Floral dip: a simplified method for *Agrobacterium*-mediated transformation of *Arabidopsis thaliana* . Plant J 16: 735–743. 10069079 10.1046/j.1365-313x.1998.00343.x

[19] Hadi MZ, Kemper E, Wendeler E, Reiss B (2002) Simple and versatile selection of *Arabidopsis* transformants. Plant Cell Reports 21: 130–135.

[20] Larkindale J, Hall JD, Knight MR, Vierling E (2005) Heat stress phenotypes of *Arabidopsis* mutants implicate multiple signaling pathways in the acquisition of thermotolerance. Plant Physiol 138: 882–897. 15923322 10.1104/pp.105.062257 PMC1150405

[21] Xin Z, Browse J (1998) Eskimo1 mutants of *Arabidopsis* are constitutively freezing-tolerant. Proc Natl Acad Sci USA 95: 7799–7804. 9636231 10.1073/pnas.95.13.7799 PMC22762

[22] Verslues PE, Agarwal M, Agarwal SK, Zhu J, Zhu JK, et al (2006) Methods and concepts in quantifying resistance to drought, salt and freezing, abiotic stresses that affect plant water status. The Plant Journal 45: 523–539. 16441347 10.1111/j.1365-313X.2005.02593.x

[23] Lim CC, Arora R, Townsend ED (1998) Comparing Gompertz and Richards functions to estimate freezing injury in *Rhododendron* using electrolyte leakage. J Amer Soc Horticult Sci 123: 246–252.

[24] Beyer WF, Fridovich I (1987) Assaying for superoxide dismutase activity: some large consequences of minor changes in conditions. Anal Biochem 161: 559–566. 3034103 10.1016/0003-2697(87)90489-1

[25] Bradford MM (1976) A rapid and sensitive method for the quantitation of microgram quantities of protein utilizing the principle of protein-dye binding. Anal Biochem 72: 248–254. 942051 10.1016/0003-2697(76)90527-3

[26] Maxwell K, Johnson G (2000) chlorophyll fluorescence- A practical guide. J Exp Bot 51: 659–668. 10938857 10.1093/jxb/51.345.659

[27] Appel RD, Bairoch A, Hochstrasser DF (1994) A new generation of information retrieval tools for biologists: the example of the ExPASy WWW server. Trends Biochem Sci 19: 258–260. 8073505 10.1016/0968-0004(94)90153-8

[28] Kelley LA, Sternberg MJE (2009) Protein structure prediction on the web: A case study using the Phyre server. Nature Protocols 4: 363–371. 19247286 10.1038/nprot.2009.2

[29] Bowles D, Lim EK, Poppenberger B, Vaistij FE (2006) Glycosyltransferases of lipophilic small molecules. Annu Rev Plant Biol 57: 567–597. 16669774 10.1146/annurev.arplant.57.032905.105429

[30] Surjus, Durand M (1996) Lipid changes in soybean root membranes in response to salt treatment. J Exp Bot 47: 17–23.

[31] Liljenberg CS (1992) The effects of water deficit stress on plant membrane lipids. Prog Lipid Res 31: 335–343. 1287669 10.1016/0163-7827(92)90012-8

[32] Baker J, Steele C, Dure L (1988) Sequence and characterization of *6 Lea* proteins and their genes from cotton. Plant Mol Biol 11: 277–291. 24272341 10.1007/BF00027385

[33] Nishiyama R, Le DT, Watanabe Y, Matsui A, Tanaka M, et al (2012) Transcriptome analyses of a salt-tolerant cytokinin-deficient mutant reveal differential regulation of salt stress response by cytokinin deficiency. PLoS One 7: e32124. 22355415 10.1371/journal.pone.0032124 PMC3280229

[34] Gill T, Kumar S, Ahuja PS, Sreenivasulu Y (2010) Over-expression of *Potentilla* superoxide dismutase improves salt stress tolerance during germination and growth in *Arabidopsis thaliana.* . J Plant Genet Trans 1: 1–10.

[35] Napoli C, Triggiani M, Palumbo G, Condorelli M, Chiariello M, et al (1997) Glycosylation enhances oxygen radical-induced modifications and decreases acetylhydrolase activity of human low density lipoprotein. Basic Res Cardiol 92: 96–105. 9166989 10.1007/BF00805570

[36] Husar S, Berthiller F, Fujioka S, Rozhon W, Khan M, et al (2011) Overexpression of the UGT73C6 alters brassinosteroid glucoside formation in *Arabidopsis thaliana* . BMC Plant Biol 11: 51. 21429230 10.1186/1471-2229-11-51 PMC3073898

[37] Lin FY, Lu QX, Xu JH, Shi JR (2008) Cloning and expression analysis of two salt and *Fusarium graminearum* stress associated UDP-glucosyltransferases genes in wheat. Yi Chuan 30: 1608–1614. 19073578 10.3724/sp.j.1005.2008.01608

[38] Heath RL, Packer L (1968) Photoperoxidation in isolated chloroplasts. I. Kinetics and stoichiometry of fatty acid peroxidation. Arch Biochem Biophys 125: 189–198. 5655425 10.1016/0003-9861(68)90654-1

[39] Chen J, Burke JJ, Xin Z, Xu C, Velten J (2006) Characterization of the *Arabidopsis* thermosensitive mutant atts02 reveals an important role for galactolipids in thermotolerance. Plant Cell Environ 29: 1437–1448. 17080965 10.1111/j.1365-3040.2006.01527.x

[40] Kingston RE, Schuetz TJ, Larin Z (1987) Heat inducible human factor that binds to a human hsp70 promoter. Mol Cell Biol 7: 1530–1534. 3600634 10.1128/mcb.7.4.1530-1534.1987 PMC365241

[41] Murakami-Murofushi K, Nishikawa K, Hirakawa E, Murofushi H (1997) Heat stress induces a glycosylation of membrane sterol in myxoamoebae of a true slime mold, *Physarum polycephalum* . J Biol Chem 272: 486–489. 8995287 10.1074/jbc.272.1.486

[42] Kunimoto S, Kobayashi T, Kobayashi S, Murakami-Murofushi K (2000) Expression of cholesteryl glucoside by heat shock in human fibroblasts. Cell Stress Chaperones 5: 3–7. 10701833 10.1043/1355-8145(2000)005<0003:EOCGBH>2.0.CO;2 PMC312902

[43] Kunimoto S, Murofushi W, Kai H, Ishida Y, Uchiyama A, et al (2002) Steryl glucoside is a lipid mediator in stress-responsive signal transduction. Cell Struct and Funct 27: 157–162. 10.1247/csf.27.157 12207046

[44] Levitt J (1980) Responses of Plants to Environmental Stress. Chilling, Freezing, and High Temperature Stresses, Ed 2. Academic Press, New York. 14.

[45] Lee JY, Joo H, Choy YH, Ha-Lee YM, Lee DH, et al (2010) Using specialized cDNA microarrays to analyze *Arabidopsis* gene expression under cold stress. J Plant Biol 53: 240–250.

[46] Grille S, Zaslawski A, Thiele S, Plat J, Warnecke D (2010) The functions of steryl glycosides come to those who wait: Recent advances in plants, fungi, bacteria and animals. Prog Lipid Res 49: 262–288. 20138912 10.1016/j.plipres.2010.02.001

[47] Houde M, Belcaid M, Ouellet F, Danyluk J, Monroy AF, et al (2006) Wheat EST resources for functional genomics of abiotic stress. BMC Genomics 7: 149. 16772040 10.1186/1471-2164-7-149 PMC1539019

[48] Warnecke D, Erdmann R, Fahl A, Hube B, Muller F, et al (1999) Cloning and functional expression of UGT genes encoding sterol glucosyltransferases from *Saccharomyces cerevisiae, Candida albicans, Pichia pastoris, and Dictyostelium discoideum* . J Biol Chem 274: 13048–13059. 10224056 10.1074/jbc.274.19.13048

[49] Schaller H (2003) The role of sterols in plant growth and development. Prog Lipid Res 42: 163–175. 12689617 10.1016/s0163-7827(02)00047-4

[50] Bouvier-Nave P, Ullmann P, Rimmele D, Benveniste P (1984) Phospholipid dependence of plant UDP-glucose sterol β-D-glucosyl transferase I. Detergent mediated delipidation by selective solubilization. Plant Sci Lett 36: 19–27.

[51] Palta JP, Whitaker BD, Weiss LS (1993) Plasma membrane lipids associated with genetic variability in freezing tolerance and cold acclimation of *Solanum* Species. Plant Physiol 103: 793–803. 12231980 10.1104/pp.103.3.793 PMC159049

[52] Patterson GW, Hugly S, Harrison D (1993) Sterols and phytyl esters of *Arabidopsis thaliana* under nomal and chilling temperatures. Phytochemistry 33: 1381–1383.

[53] DeBolt S, Scheible WR, Schrick K, Auer M, Beisson F, et al (2009) Mutations in UDP-Glucose:sterol glucosyltransferase in *Arabidopsis* cause transparent testa phenotype and suberization defect in seeds. Plant Physiol 151: 78–87. 19641030 10.1104/pp.109.140582 PMC2735980

[54] Lu CM, Qiu NM, Lu QT, Wang BS, Kuang TY, et al (2002) Does salt stress lead to increased susceptibility of photosystem II to photoinhibition and changes in photosynthetic pigment composition in halophyte *Suaeda salsa* grown outdoors? Plant Sci 163: 1063–1068.

[55] Silva E, Ribeiro R, Silva S, Viegas R, Silveira J (2011) Salt stress induced damages on the photosynthesis of physic nut young plants. Sci Agric 68: 62–68.

[56] Kalaji HM, Govindjee, Bosa K, Koscielniak J, Zuk-Golaszewska K (2011) Effects of salt stress on photosystem II efficiency and CO2 assimilation of two Syrian barley landraces. Environ Exper Bot 73: 64–72.

[57] Cui L, Li J, Fan Y, Xu S, Zhang Z (2006) High temperature effects on photosynthesis, PSII functionality and antioxidant activity of two *Festuca arundinacea* cultivars with different heat susceptibility. Botanical Studies 47: 61–69.

[58] Sharkey T (2005) Effects of moderate heat stress on photosynthesis: importance of thylakoid reactions, rubisco deactivation, reactive oxygen species, and thermotolerance provided by isoprene. Plant Cell Environ 28: 269–277.

[59] Asada K (1999) The water-water cycle in chloroplasts: Scavenging of active oxygens and dissipation of excess photons. Annu Rev Plant Physiol Plant Mol Biol 50: 601–639. 15012221 10.1146/annurev.arplant.50.1.601

