# Supplementary figures and images for: Overexpression of WsSGTL1 Gene of Withania somnifera Enhances Salt Tolerance, Heat Tolerance and Cold Acclimation Ability in Transgenic Arabidopsis Plants
Source: PLoS One. 2013 Apr 30;8(4):e63064. doi: 10.1371/journal.pone.0063064 (PMC3639950; doi:10.1371/journal.pone.0063064)

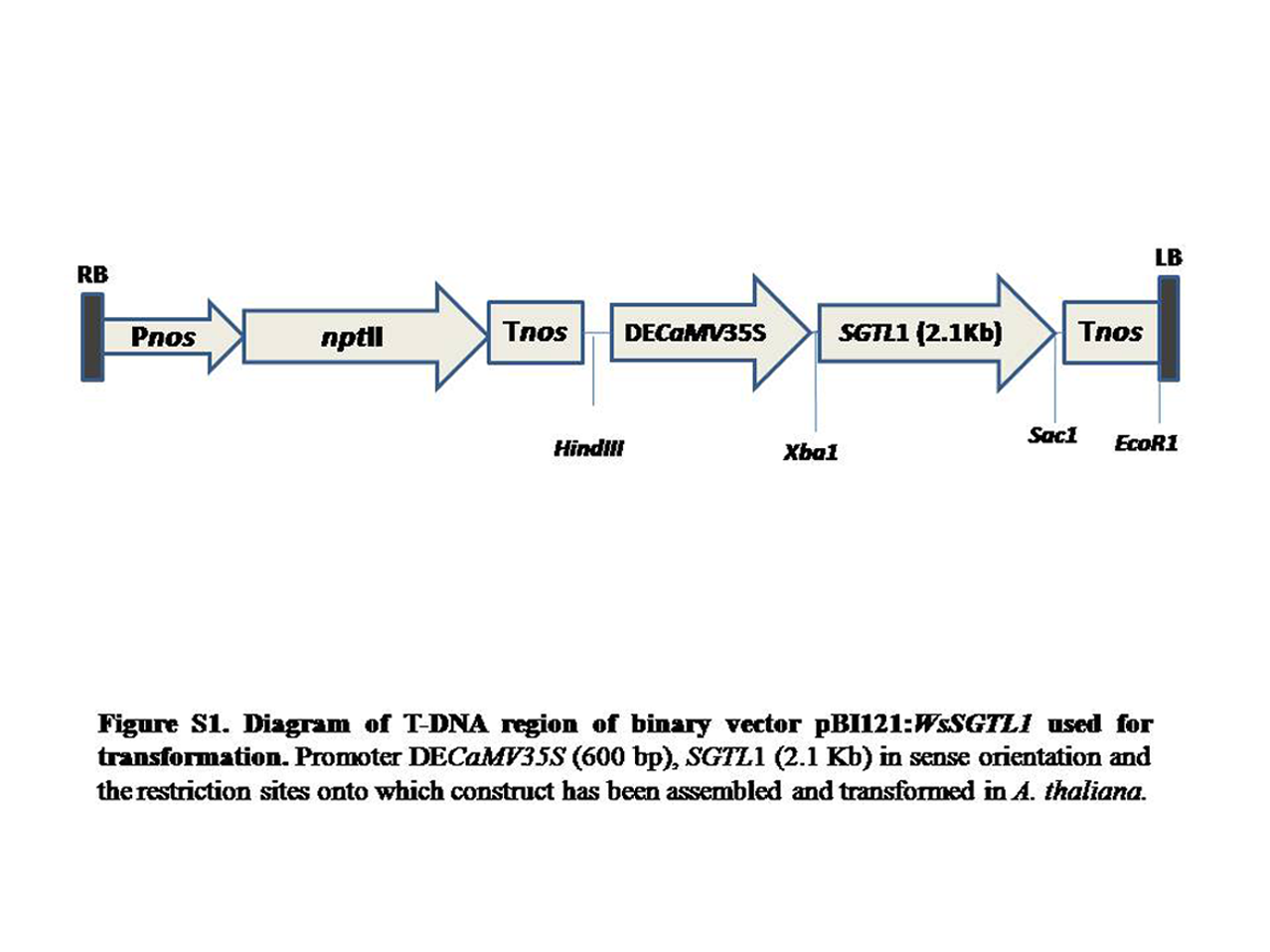

Supplement: Figure S1 — Schematic diagram showing T-DNA region of binary vector pBI121 used for transformation. The WsSGTL1 gene was inserted at the XbaI-SacI site in sense orientation. Promoter DECaMV35S (600 bp), WsSGTL1 (2.1 Kb) and the restriction sites were assembled and transformed in A. thaliana. (TIF) [file pone.0063064.s001.tif]

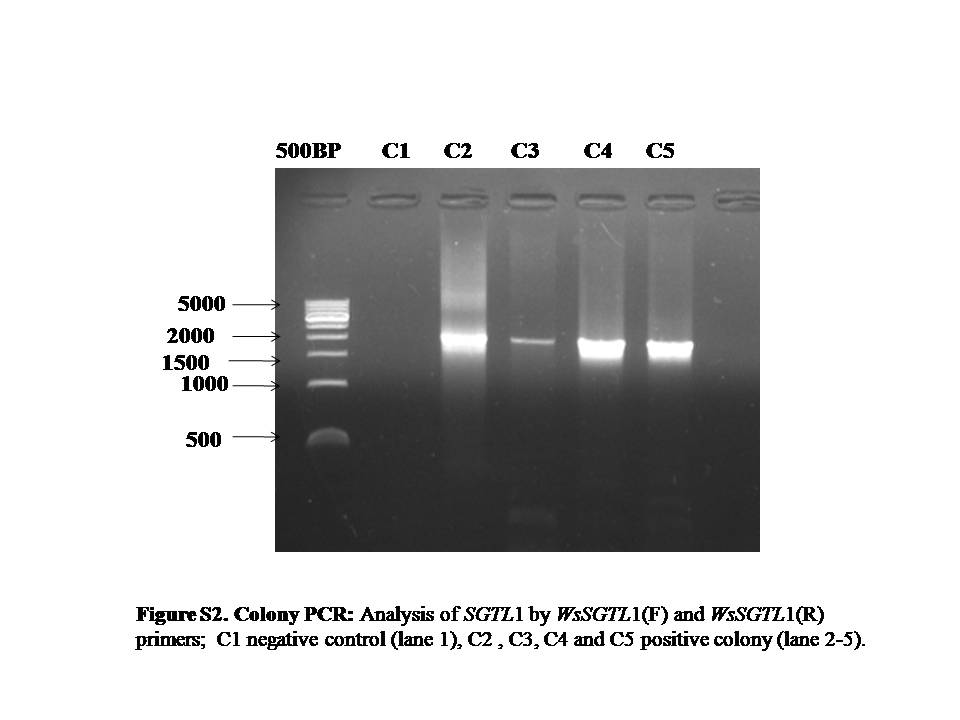

Supplement: Figure S2 — Colony PCR: Analysis of WsGTL1 by WsSGTL1 (F) and WsSGTL1(R) primers; C1 negative control (lane 1), C2, C3, C4 and C5 positive colony (lane 2–5). (TIF) [file pone.0063064.s002.tif]

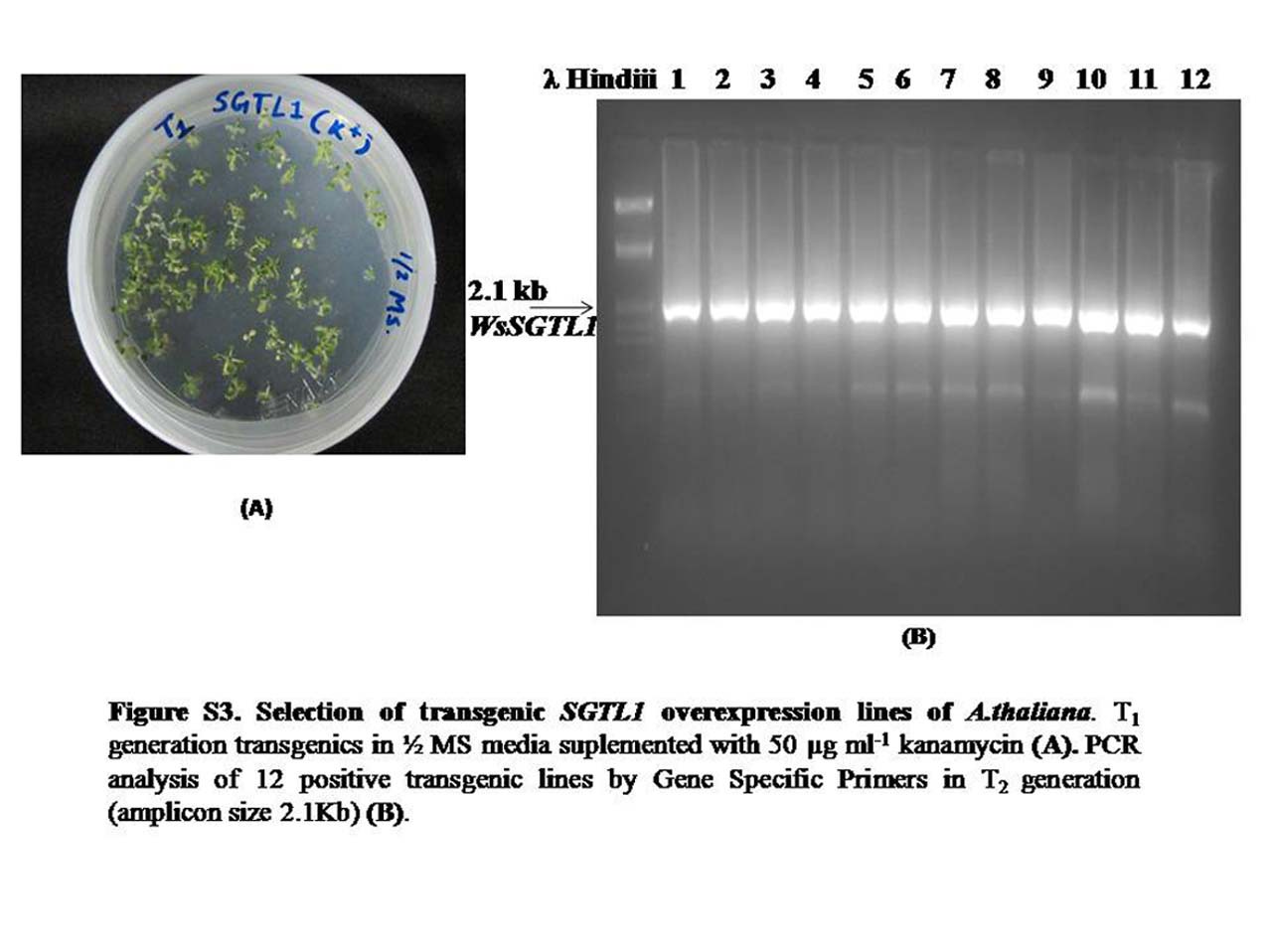

Supplement: Figure S3 — Selection of transgenic WsSGTL1 overexpression lines of A.thaliana. (A) T1 generation transgenics in ½ MS media supplemented with 50 µg ml−1 kanamycin. (B) PCR analysis of 12 positive transgenic lines by Gene Specific Primers in T2 generation (amplicon size 2.1 Kb). (TIF) [file pone.0063064.s003.tif]

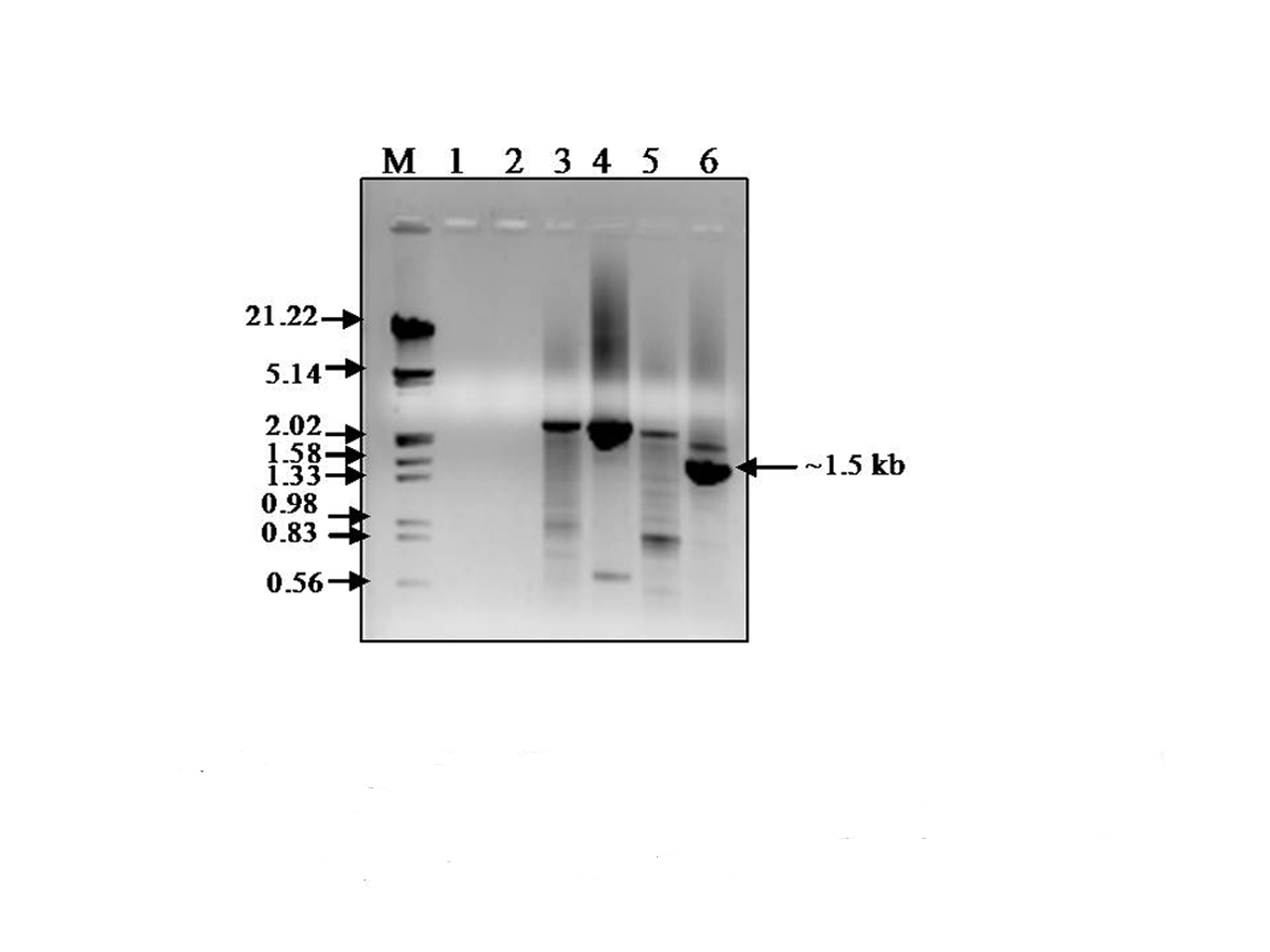

Supplement: Figure S4 — A Genome walking PCR with adaptor primers and gene specific primers. PCR products from non template control (lane 1), nested control (lane 2), AP1+GSP1 (lane 3), AP2+GSP2 (lane 4), AP1+GSP3 (lane 5) and AP2+GSP4 (lane 6) are shown. M;λ HindIII/EcoRI double digested DNA molecular weight markers. B. Promoter sequence of WsSGTL1. Nucleotide sequence of WsSGTL1 promoter including intronic region (shown as red color) and overlapping sequence of SGTL1 (shown as green color). (ZIP) [file pone.0063064.s004.zip › Figure S4 A in File S4.tif]

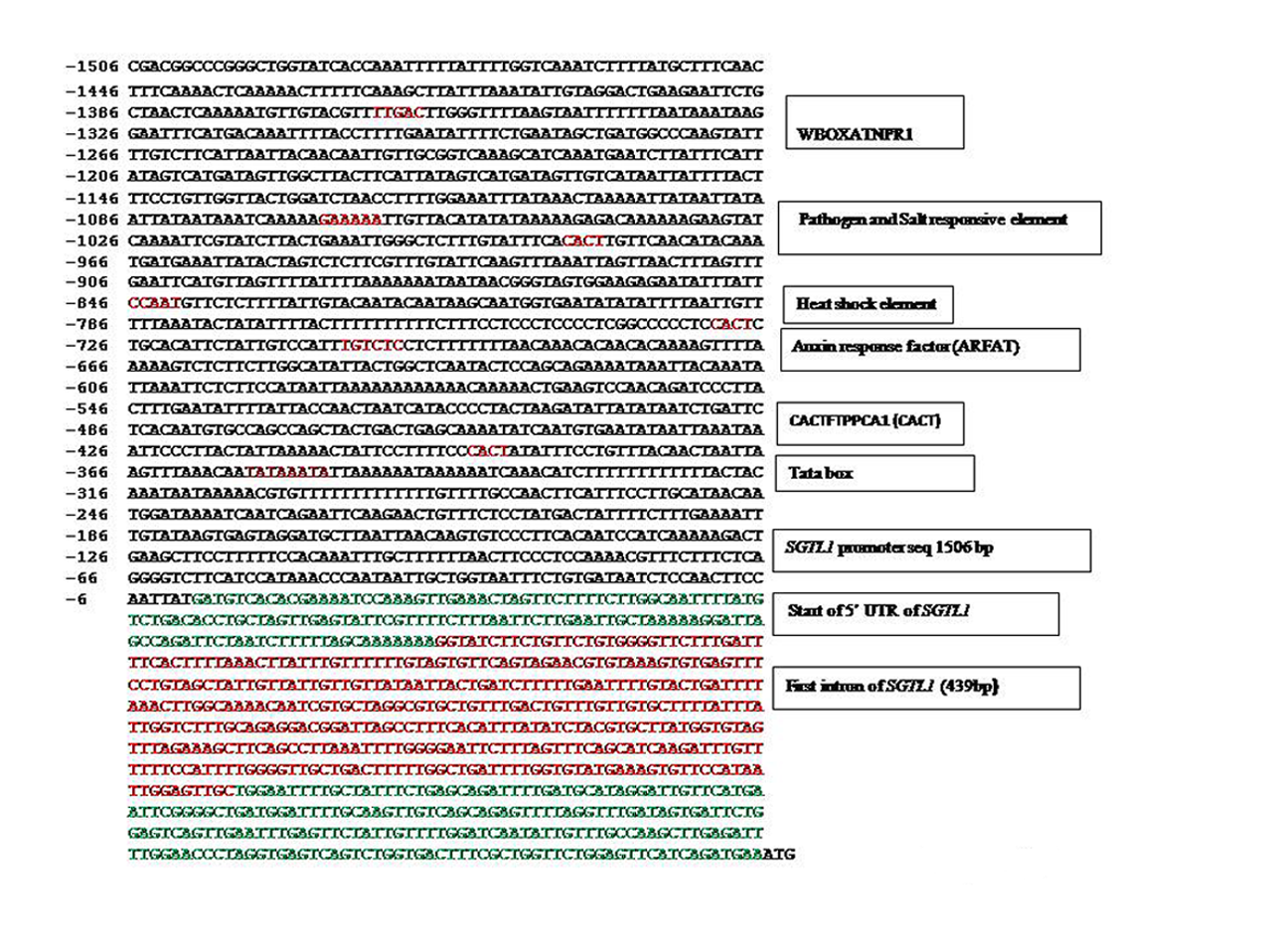

Supplement: Figure S4 — A Genome walking PCR with adaptor primers and gene specific primers. PCR products from non template control (lane 1), nested control (lane 2), AP1+GSP1 (lane 3), AP2+GSP2 (lane 4), AP1+GSP3 (lane 5) and AP2+GSP4 (lane 6) are shown. M;λ HindIII/EcoRI double digested DNA molecular weight markers. B. Promoter sequence of WsSGTL1. Nucleotide sequence of WsSGTL1 promoter including intronic region (shown as red color) and overlapping sequence of SGTL1 (shown as green color). (ZIP) [file pone.0063064.s004.zip › Figure S4 B in File S4.tif]

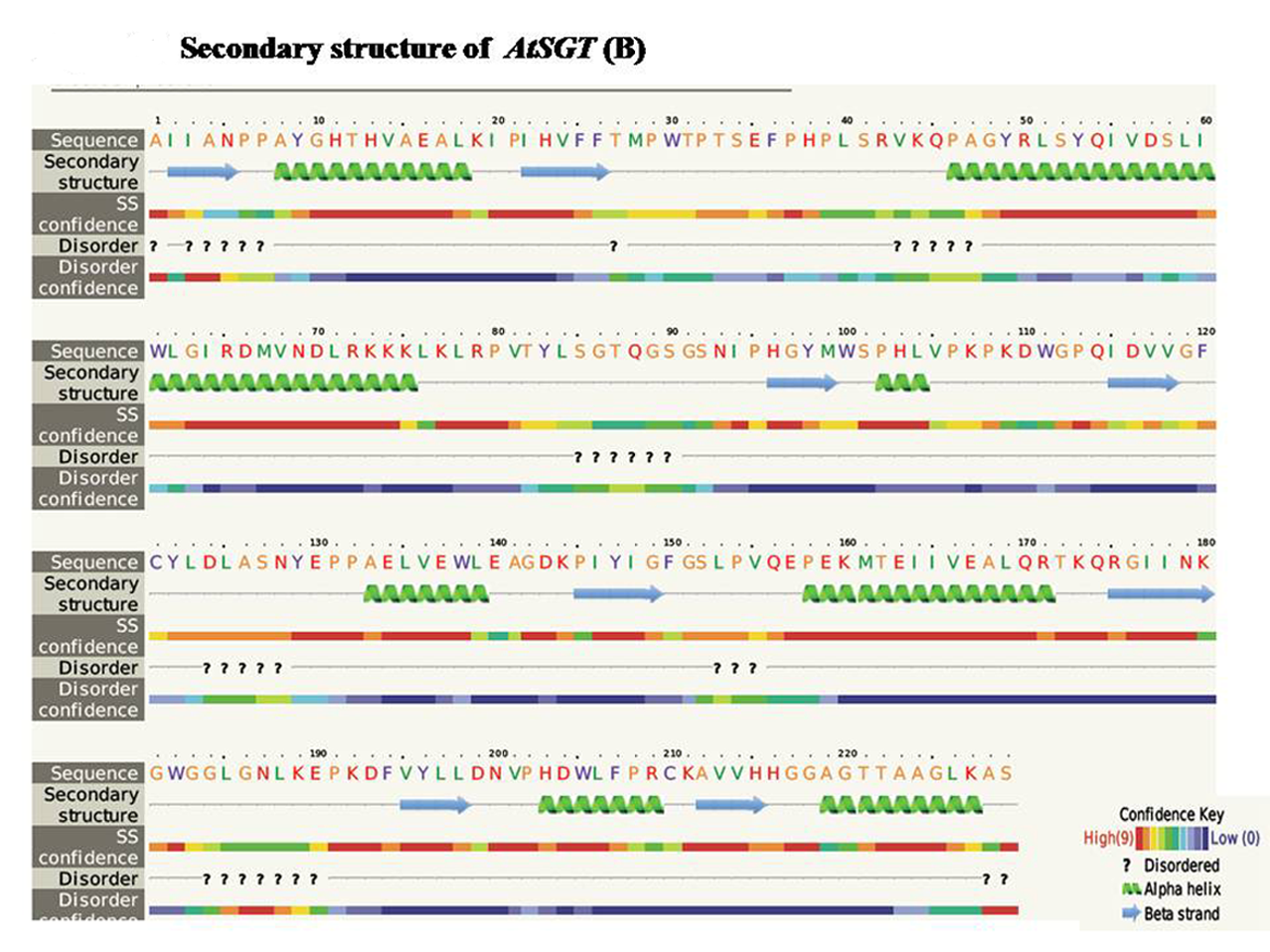

Supplement: Figure S5 — Secondary structure and alignments (A) Secondary structure of WsSGTL1 . (B) Secondary structure of AtSGT . (C) Alignment of WsSGTL1 and AtSGT. (ZIP) [file pone.0063064.s005.zip › Figure S5 B in File S5.tif]

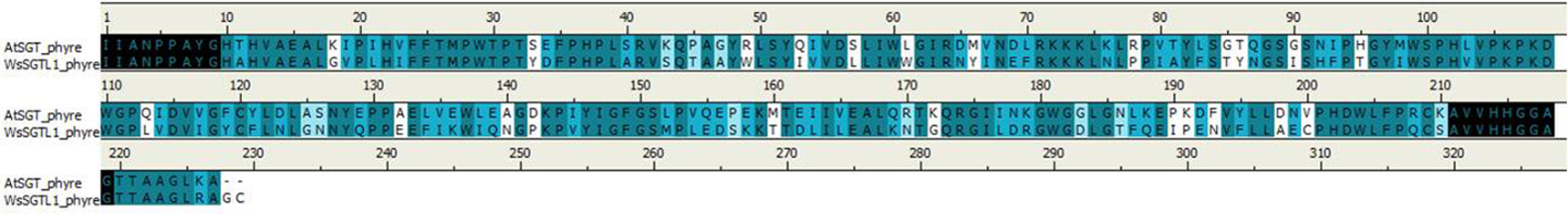

Supplement: Figure S5 — Secondary structure and alignments (A) Secondary structure of WsSGTL1 . (B) Secondary structure of AtSGT . (C) Alignment of WsSGTL1 and AtSGT. (ZIP) [file pone.0063064.s005.zip › Figure S5 C in File S5.tif]

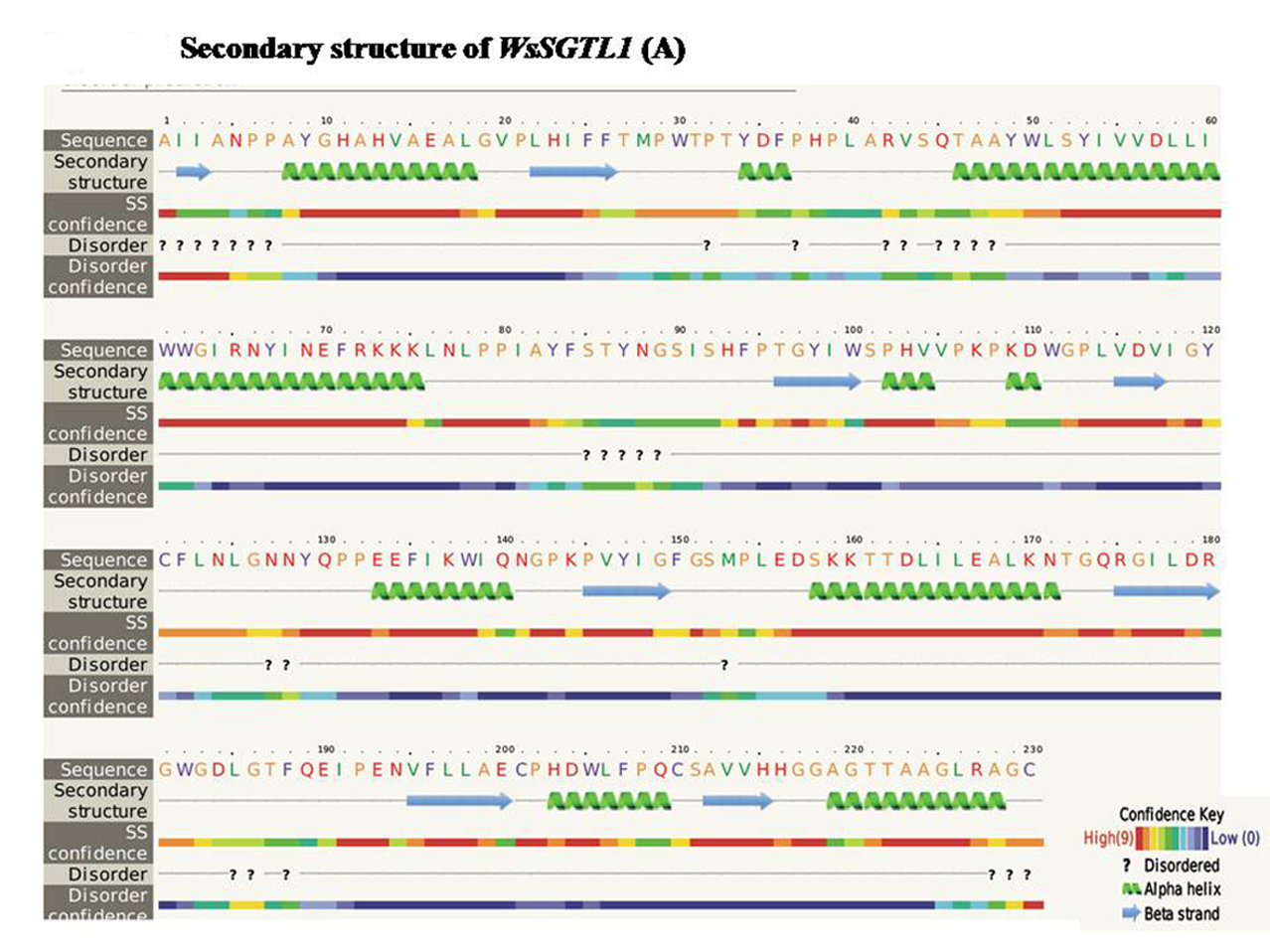

Supplement: Figure S5 — Secondary structure and alignments (A) Secondary structure of WsSGTL1 . (B) Secondary structure of AtSGT . (C) Alignment of WsSGTL1 and AtSGT. (ZIP) [file pone.0063064.s005.zip › Figure S5 A in File S5.tif]

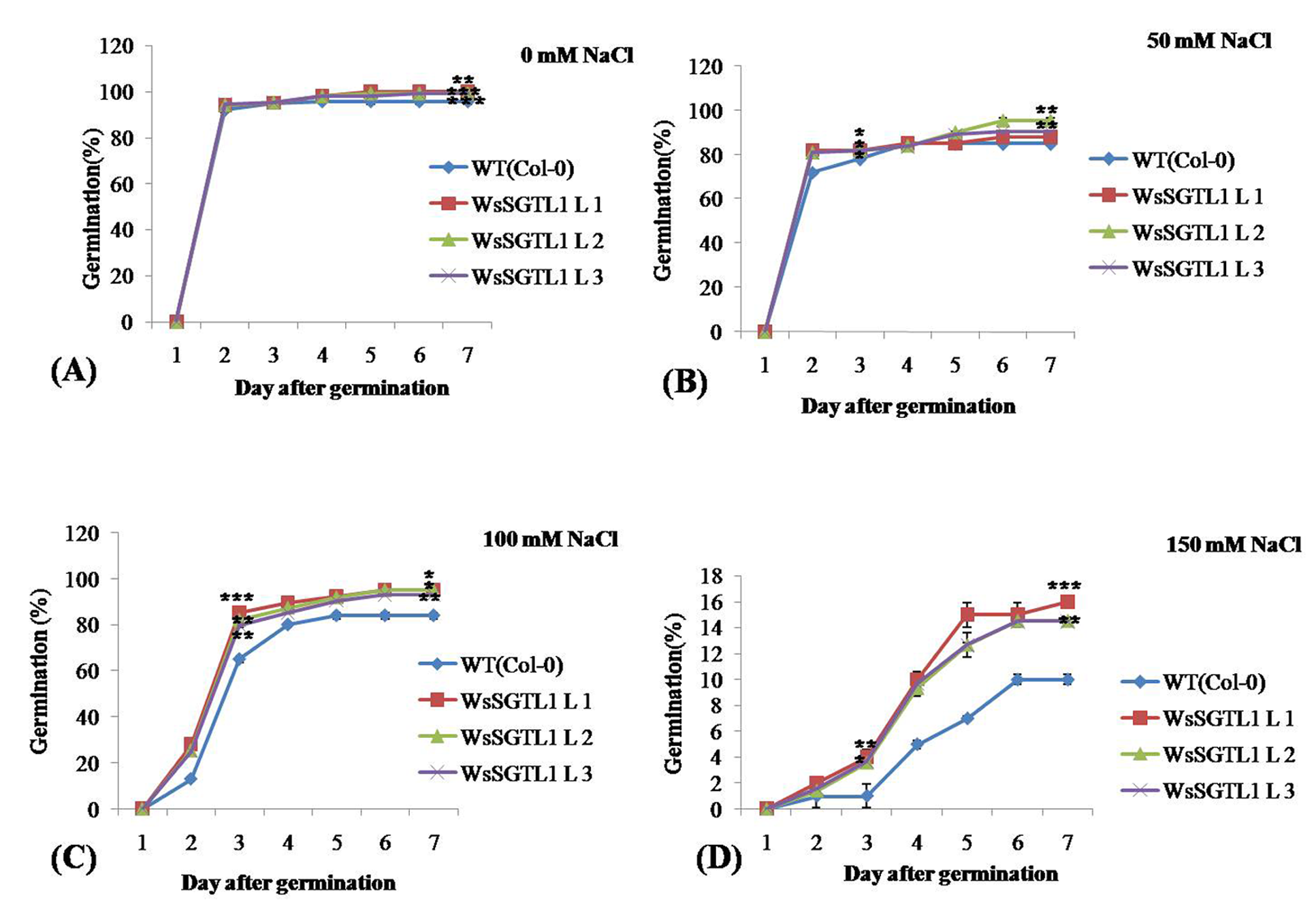

Supplement: Figure S6 — Germination rate and early seedling development of both WT and overexpression lines of A.thaliana under salt stress. (A) Percentage of germinating seeds of WT and WsSGTL1 overexpression lines (L1, L2 and L3) of T3 plants grown on MS medium. (B) Supplemented with 50 mM NaCl. (C) With 100 mM NaCl. (D) With 150 mM NaCl. Data of germination recorded after 7 days of germination. Values are percentage germination ± SE, n = 60, (**) for P≤0.01 (***) for P≤0.001 or 0.005 significantly different from the control (t-test) from three independent experiments. (TIF) [file pone.0063064.s006.tif]

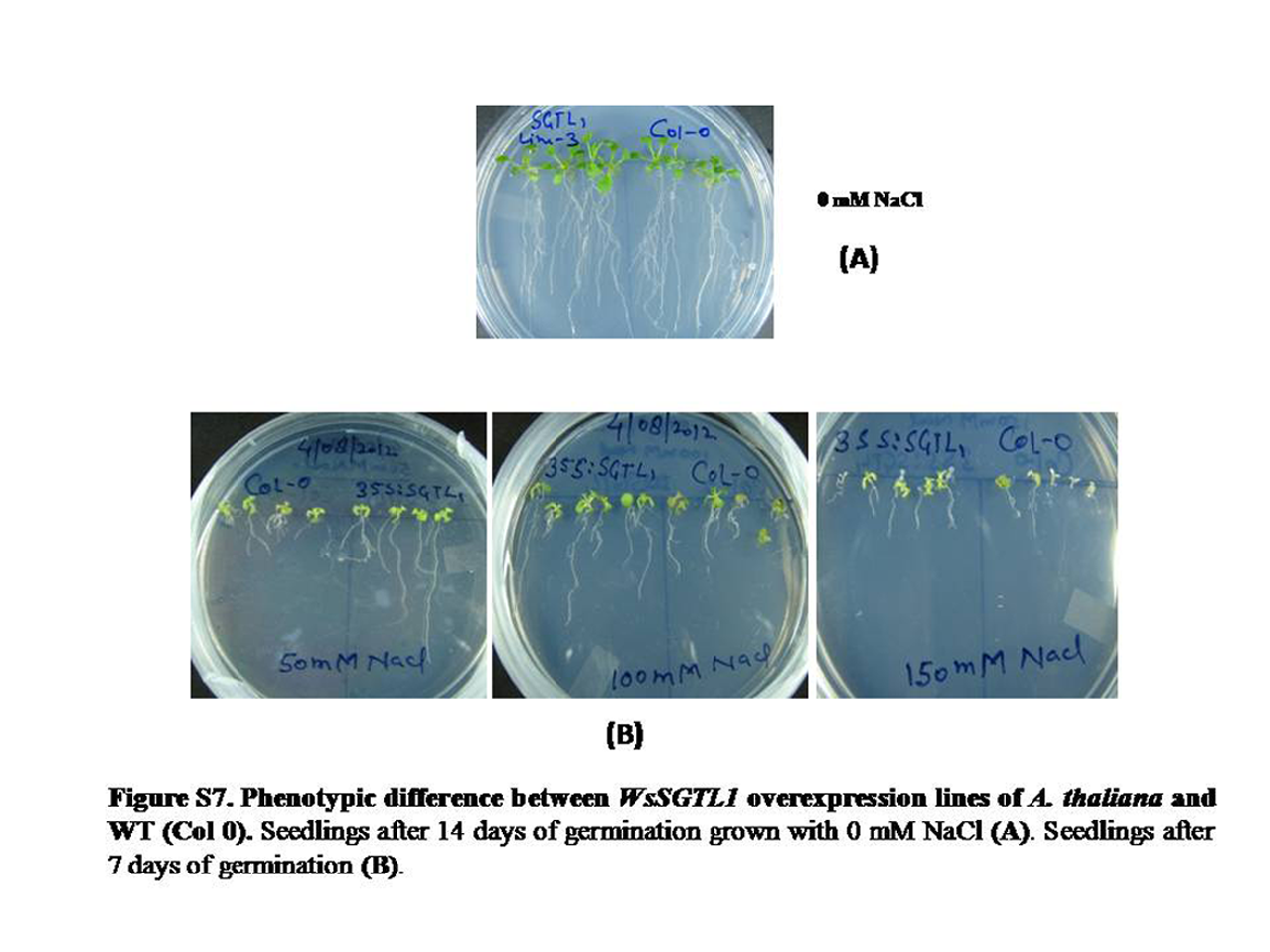

Supplement: Figure S7 — Phenotypic differences between WsSGTL1 overexpression lines of A. thaliana and WT (Col 0). (A) Seedlings after 14 days of germination grown with 0 mM NaCl. (B) Seedlings grown after 7 days of germination (from L to R); with 50 mM NaCl; with 100 mM NaCl; with 150 mM NaCl. (TIF) [file pone.0063064.s007.tif]

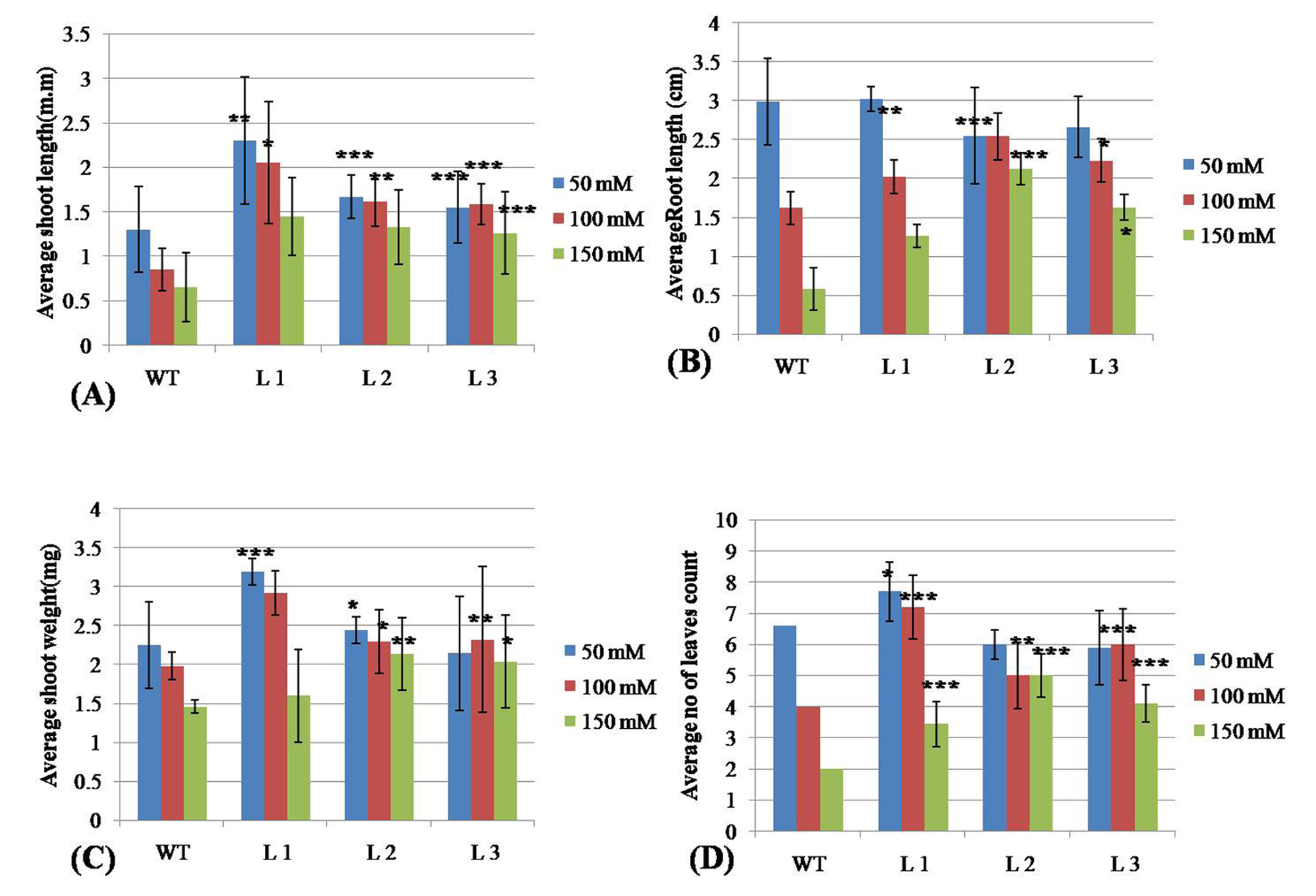

Supplement: Figure S8 — Data of phenotypic comparison of WT plants and WsSGTL1 overexpression lines of A. thaliana after 14 days of germination. Comparison of shoot length, root length, shoot weight and leaf count of WT and WsSGTL1 overexpression lines of A. thaliana grown on MS medium supplemented with 50 mM NaCl, 100 mM NaCl and 150 mM NaCl. (A) Average shoot length.(B) Average root length. (C) Average shoot weight. (D) Average number of leaves. Values are mean ± SE, n = 10, (*) for P≤0.05, (**) for P≤0.01, (***) for P≤0.001 or 0.005 significantly different from the control (t-test). (TIF) [file pone.0063064.s008.tif]

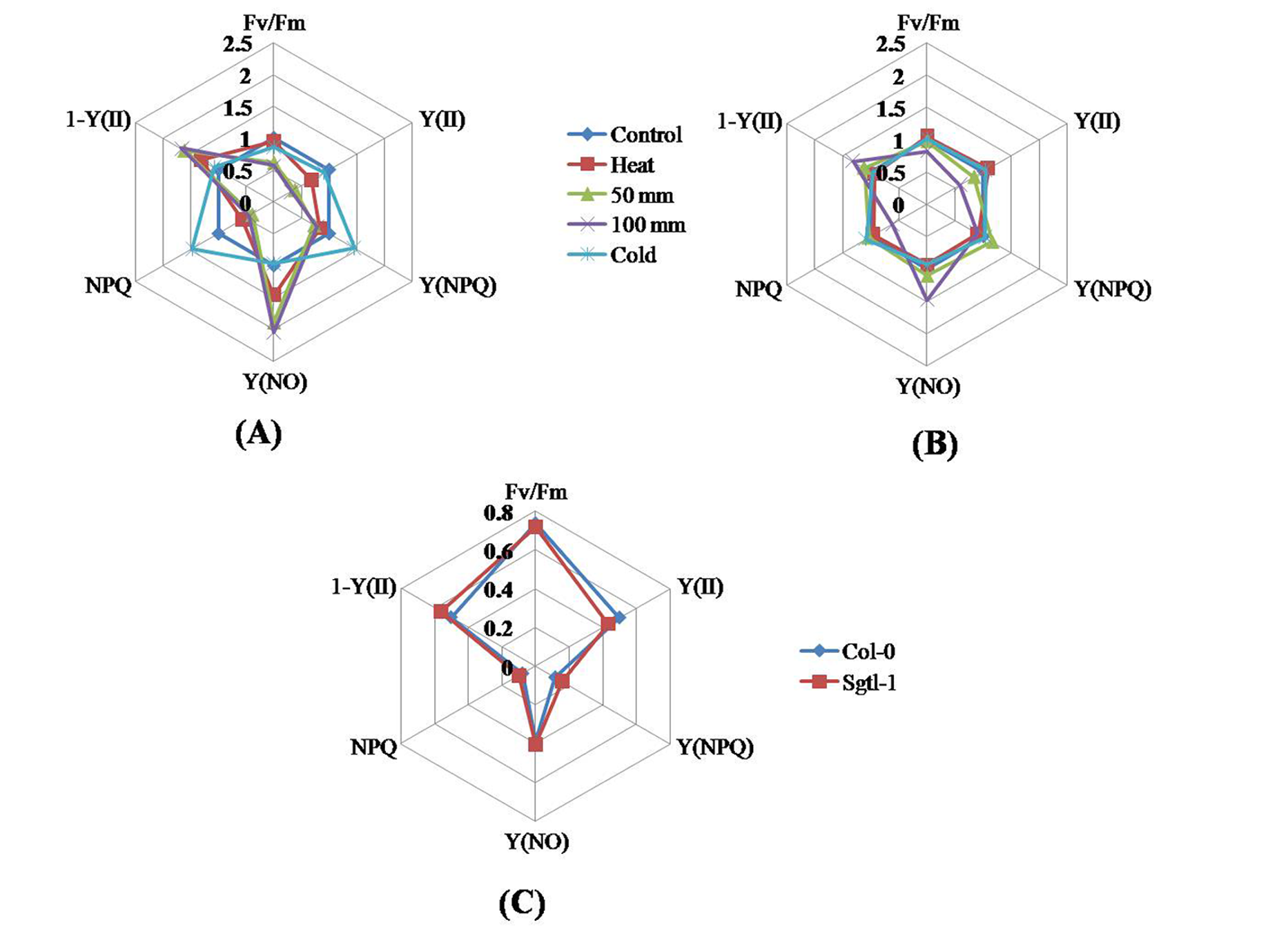

Supplement: Figure S9 — Spider plots of chlorophyll fluorescence. Spider plots showing relative changes of mean values of selected fluorescence parameters of maximum photosynthetic efficiency (Fv/Fm), photosynthetic yield Y(II), excitation pressure 1-Y(II), total nonphotochemical quenching (NPQ), regulated heat dissipation Y (NPQ), and unregulated heat dissipation Y (NO) under different abiotic stress of both the genotype of A. thaliana. (A) Wild type plants(Col-0). (B) WsSGTL1 transgenic lines. (C) Both WT and transgenic lines under normal growth conditions. Data from normal growth (control condition) were adjusted to 1.0, and all other parameters were calculated as fold-changes. (TIF) [file pone.0063064.s009.tif]

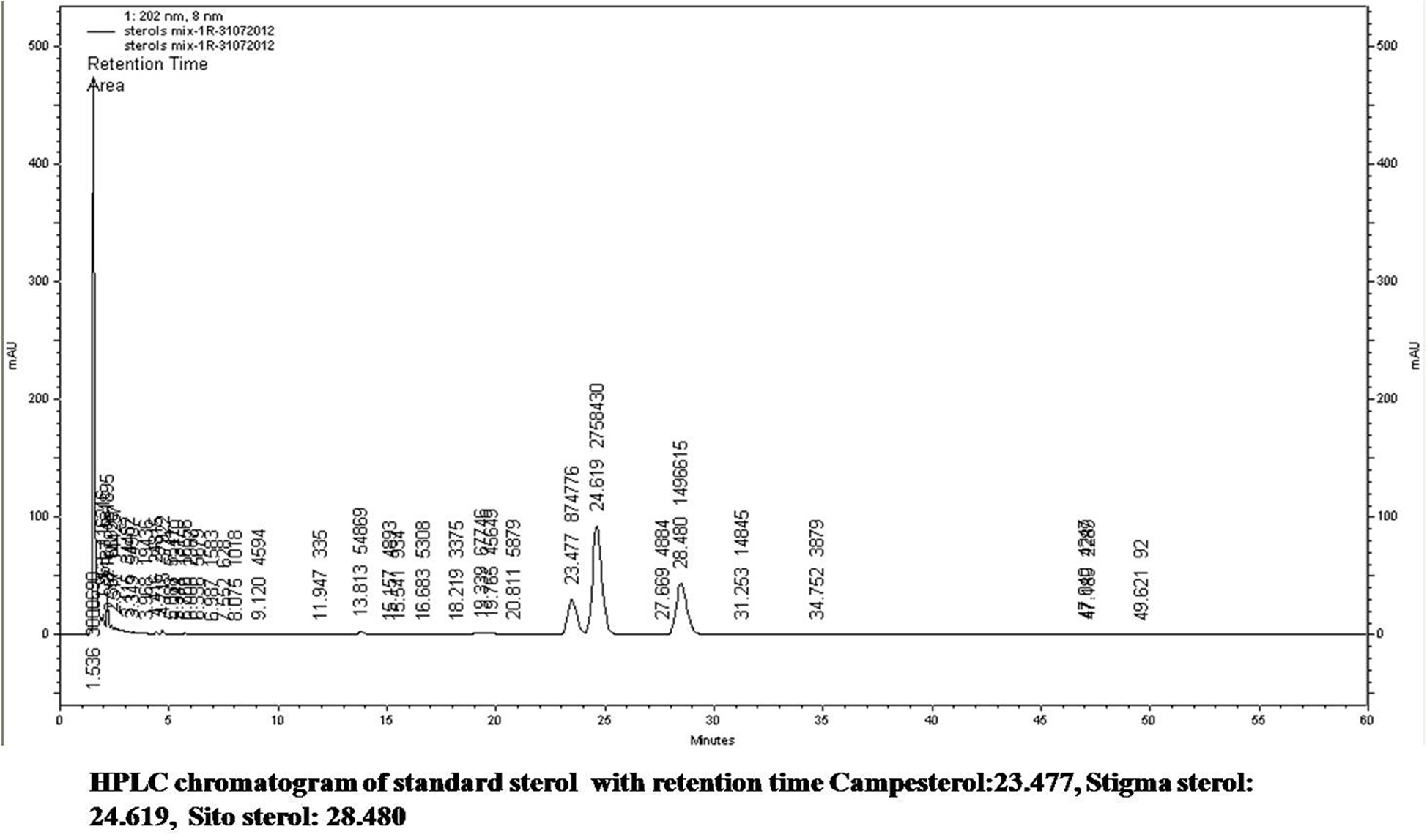

Supplement: Figure S10 — HPLC chromatogram of standard sterol compounds. HPLC chromatogram of standard sterol with retention time Campesterol: 23.477, Stigma sterol: 24.619, Sito sterol: 28.480 (TIF) [file pone.0063064.s010.tif]

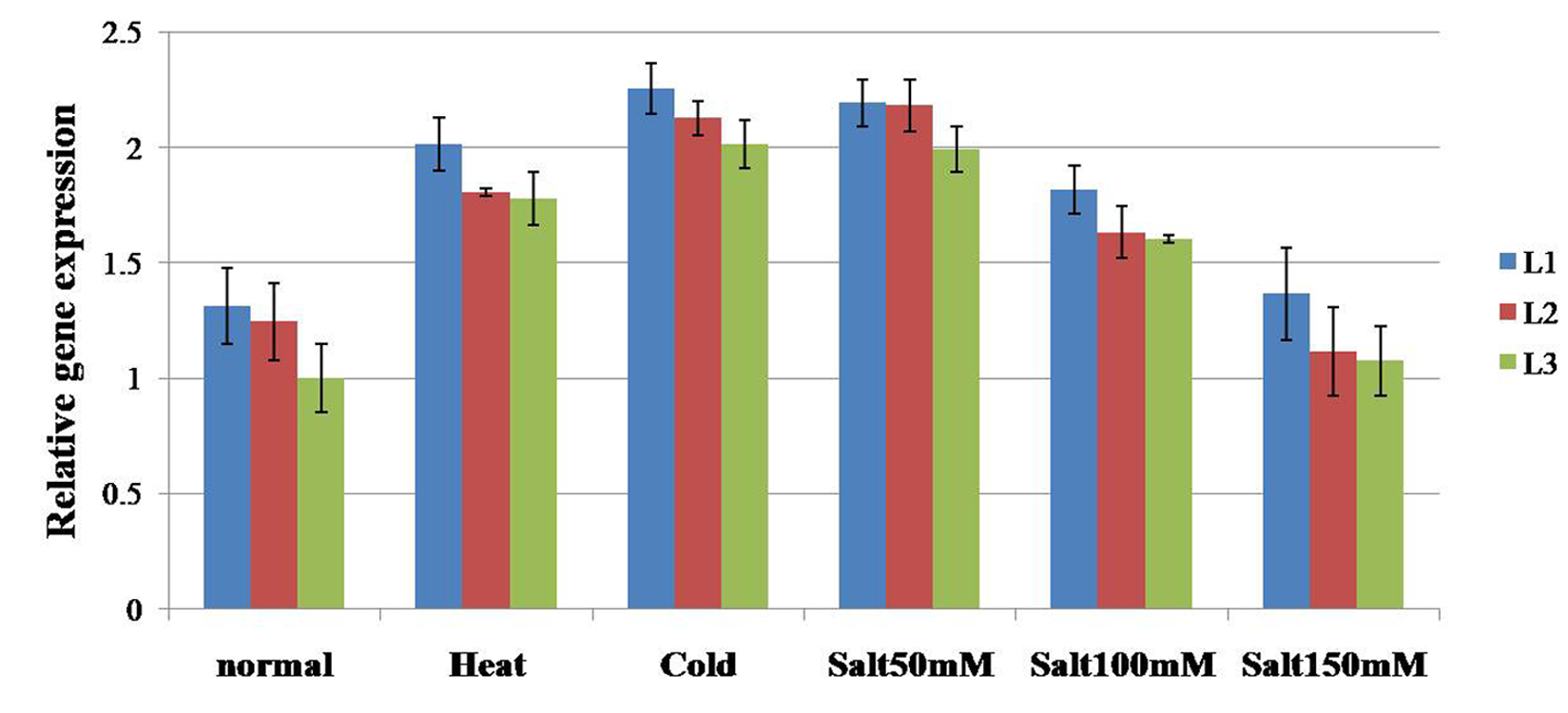

Supplement: Figure S11 — Relative expression of WsSGTL1 through real time PCR. Relative expression of WsSGTL1 in transgenic A.thaliana regulated by CaMV35S promoter under salt, heat and cold stress. Plants were treated with 0, 50, 100 or 150 mM NaCl for 24 h. WsSGTL1 expression was not detected in WT plants grown under both with or without salt stress. While it was markedly increased in L1 and L2 lines and slightly increased in L3 line under salt stress. At 150 mM NaCl stress, expression level was low. The seedlings were exposed to 42°C for 4 h to provide heat stress, while at 4°C for 24 h for cold stress. The expression of L1 and L2 was remarkably increased as compared to non treated plants. (TIF) [file pone.0063064.s011.tif]

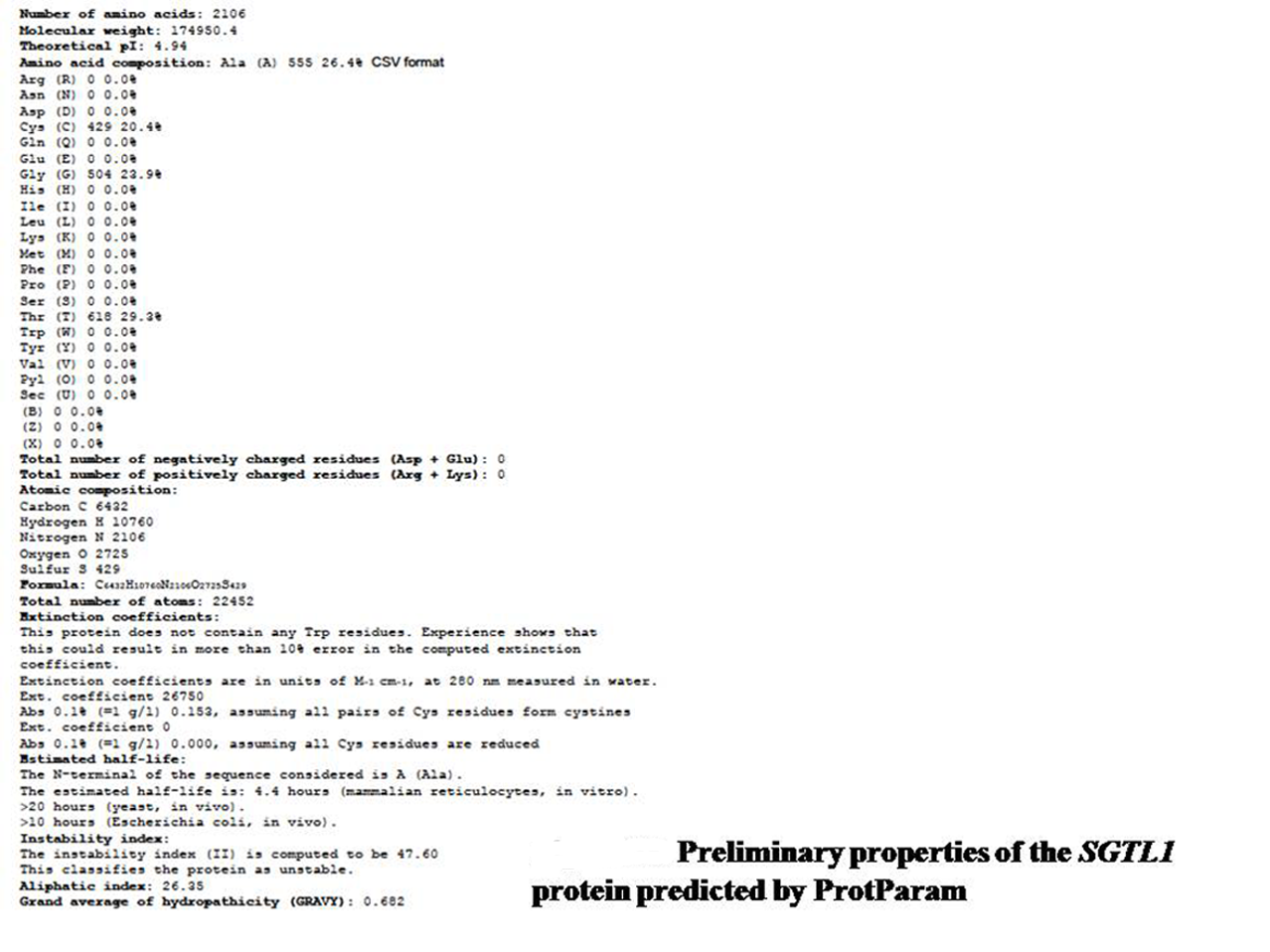

Supplement: Figure S12 — Preliminary properties of the WsSGTL1 protein predicted by ProtParam. (TIF) [file pone.0063064.s012.tif]

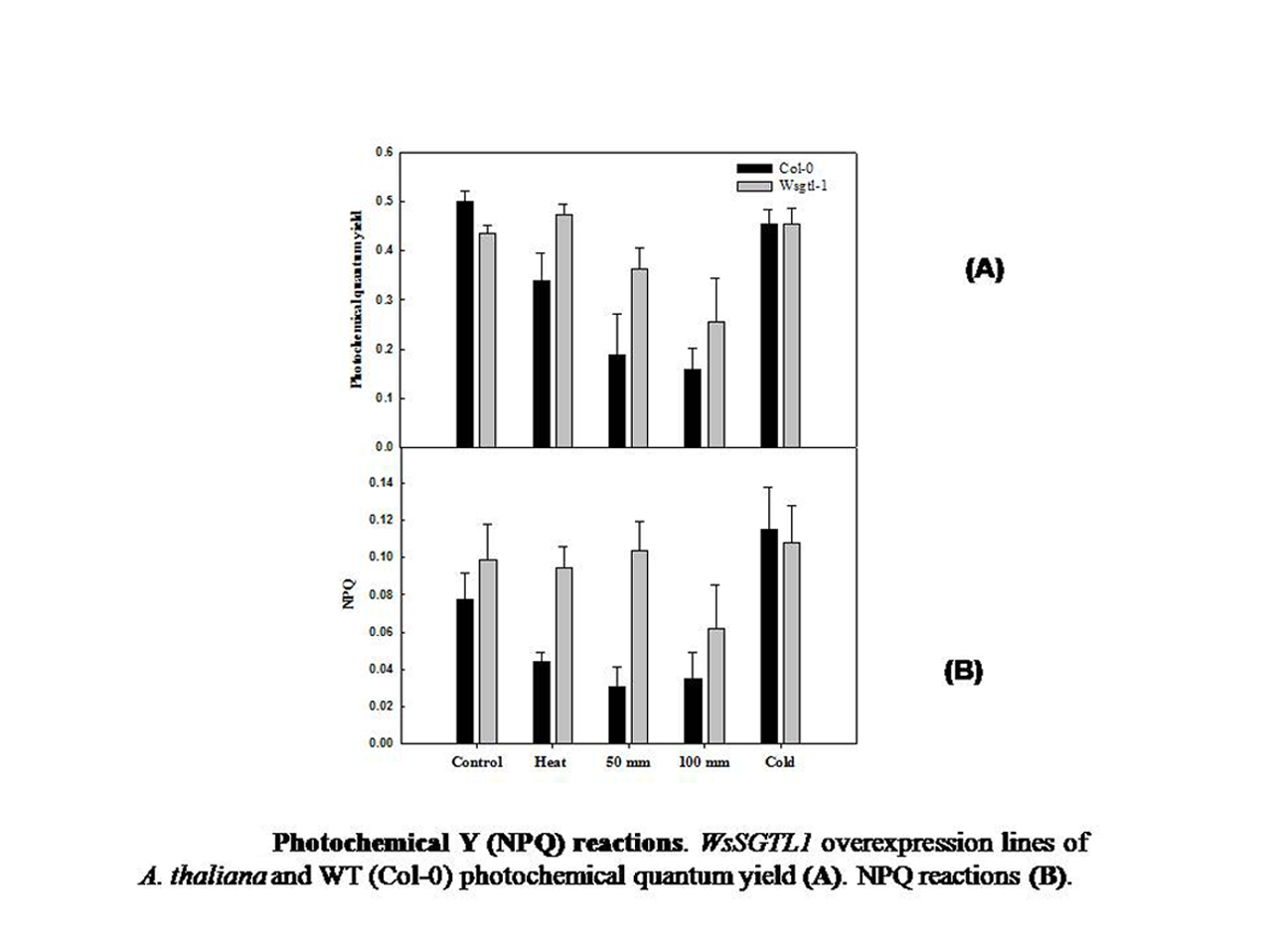

Supplement: Figure S13 — Photosynthetic yield [Y (II)] and total heat dissipation (NPQ) under different abiotic stress conditions. (A) In WT and transgenic lines of A.thaliana, photosynthetic yield decreased at 50 and 100 mM NaCl but in WT its values decreased significantly due to stress (P≤0.01). (B) Total heat dissipation in WT decreased significantly (P≤0.01) but not under cold stress, whereas transgenic lines were much affected during salt, heat and cold stress. (TIF) [file pone.0063064.s013.tif]
