# Supplementary material for: Overexpression of WsSGTL1 Gene of Withania somnifera Enhances Salt Tolerance, Heat Tolerance and Cold Acclimation Ability in Transgenic Arabidopsis Plants
Source: PLoS One. 2013 Apr 30;8(4):e63064. doi: 10.1371/journal.pone.0063064 (PMC3639950; doi:10.1371/journal.pone.0063064)
Supplement: Table S1 — List of primers used in present study. (DOCX) [file pone.0063064.s014.docx]

**Table S1. List of primers used in study.**

| **Name of Primer** | **Sequence of Primers** | **Forward/Reverse** | **Use and specification** |
| --- | --- | --- | --- |
| *WsSGTl1*(F1) | GC***TCTAGA***ATGGACAGTAATGGGCATAATGGCA | Forward primer with Xba1 site | For cloning of *WsSGTL1*gene in pBI121 vector |
| *WsSGTL1*(R1) | ATAGC***GAGCTC***TTAAGACCCACAAGGC | Reverse primer with Sac1 site(for cloning) | For cloning of *WsSGTL1*gene in pBI121 vector |
| *WsSGTL1*(F2) | ATGGACAGTAATGGGCATAATGGCACTAC | Gene specific forward primer(for cloning) | For cloning of *WsSGTL1*gene in pBI121 vector |
| *WsSGTL1*(R2) | TTAAGACCCACAAGGCAGGCAACAGATT | Gene specific reverse primer (for cloning) | For cloning of *WsSGTL1*gene in pBI121 vector |
| M13(F)- | GTAAAACGACGGCCAGT | Sequencing primer forward | Sequencing analysis of pTZ: *WsSGTL1* construct |
| M13(R) | CAGGAAACAGCTATGAC | Sequencing primer reverse | Sequencing analysis of pTZ: *WsSGTL1* construct |
| *CaMV*(F) | GTAAGGGATGACGCACAATCC | *CaMV35 S* forward primer | Positive selection of transgene in pBI121:WsSGTL1 in transformed vector. |
| *NosT*(R)- | GGACTCTAATCATAAAAACCC | *Nos*T reverse primer | Positive selection of transgene in pBI121:WsSGTL1 in transformed vector |
| *Ubiquitin*(F) (AJ309010) | GAAGCAGCTCGAGGATGGAA | Fwd primer for RT PCR of *Ubiquitin* | Housekeeping genes of tobacco for real time PCR of *W.somnifera* |
| *Ubiquitin*(R) (AJ309010) | CCACGGAGACGGAGGACAA | Rev.primer for RT PCR of *Ubiquitin* | Housekeeping genes of tobacco for real time PCR of *W.somnifera* under salt stress. |
| *Actin2(*F)  (At3g18780) | TCCCTCAGCACATTCCAGCAGAT | Fwd primer for RT PCR of *Actin* | Housekeeping genes of *A.thaliana* for real time PCR of different abiotic stress. |
| *Actin2(*R)  (At3g18780) | AACGATTCCTGGACCTGCCTCATC | Rev. primer for RT PCR of *Actin* | Housekeeping genes of *A. thaliana* for real time PCR of different abiotic stress. |
| *SGTL1*FARN | GACAGGACCAGTGATGTTGATTC | Forward primer for RT-PCR of *SGTL1* | Reverse transcriptase PCR analysis of *WsSGTL1* |
| *SGTL1*REVN | GCTCGAGGTGGAACACTAGAAC | Rev. primer for RT-PCR of *SGTL1* | Reverse transcriptase PCR analysis  of *WsSGTL1* |
| ***SGTL1***RTNF | GACAGTAATGGGCATAATGGCAC | New Forward primer for RT-PCR | Reverse transcriptase PCR analysis of WsSGTL1 |
| *SGTL1*RTNR | ACTTGTCCTCCAGCCATCTAATTC | New Rev. primer for RT-PCR of *SGTL1* | Reverse transcriptase PCR analysis of WsSGTL1 |
| *SGTL1*re F | GCCGAGTGCCCTCATGATT | Forward primer for  qRT-PCR of *SGTL1* | For quantitative real time PCR analysis of transgene expression |
| *SGTL1*re R | GTTGGACACCCAGCACGTAGTC | Reverse primer for  qRT-PCR of *SGTL1* | For quantitative real time PCR analysis of transgene.expression |
| AT3G12580 (*AtHsp70*)F | GGTATACCACCTGCTCCACG | Forward primer | For reverse transcriptase PCR analysis Hsp70under Heat stress |
| AT3G12580 (*AtHsp70*)R | CTTGTCCTCAGCCGACACAT | Reverse primer | For reverse transcriptase PCR analysis Hsp70under Heat stress |
| AT5G06760  (*LEA4-5*)F | GGAAAAGGCGGAGAAGATGA | *LEA 4-5* (late embryogenesis abundant protein 4-5) forward | For reverse transcriptase PCR analysis under Salt stress condition |
| AT5G06760  (*LEA4-5*)R | TTGTGCTGACGCGTTTCTCT | *LEA 4-5* (late embryogenesis abundant protein 4-5) Reverse | For reverse transcriptase PCR analysis under Salt stress condition |
| *RD29A* (F) | CGGCGGTTTAGGAGCTCCGTTG | Dehydration responsive element accession no.(D13044.1 | For reverse transcriptase PCR analysis under Cold stress condition. |
| *RD29A* (R) | CCGTCAAATCCCGTCGGCACA | Dehydration responsive element accession no.(D13044.1) | For reverse transcriptase PCR analysis under Cold stress condition. |
| *RD29B* (F) | AAGTTCACGGCGCCACCAGG | Dehydration responsive element accession no.(D13044.2) | For reverse transcriptase PCR analysis under Cold stress condition. |
| *RD29B* (R) | CCGTTACACCACCTCTCACGGC | Dehydration responsive element accession no.(D13044.2) | For reverse transcriptase PCR analysis under Cold stress condition. |
| *Hsp90(F)* | GGATTGTGGACTCTCCCTGC | Heat shock protein (AT5G52640.1) | For reverse transcriptase PCR analysis under Heat stress condition |
| *Hsp90(R)* | GCTGCTATCTCTCAACGCCT | Heat shock protein (AT5G52640.1) | For reverse transcriptase PCR analysis under Heat stress condition |
| *SOS3(F)* | GGAGGAATCTCTTCGCTG | Salt Overlay sensitive (AF192886) | For reverse transcriptase PCR analysis under Salt stress condition |
| *SOS3(R)* | CACGAAAGCCTTATCCACC | Salt Overlay sensitive (AF192886 | For reverse transcriptase PCR analysis under Salt stress condition |
| *SGTL1* Promoter( F) | CACCCGACGGCCCGGGCTGGTATC | Internal promoter forward primer | Internal primer of WsSGTL1  Promoter |
| *SGTL1* Promoter (R) | TTCATCTGAACTCCAGAACCA | Internal promoter reverse primer | Internal primer of WsSGTL1 Promoter |
| Adaptor primer (AP1)* | GTAATACGACTCACTATAGGGC | Adapter primer for cloning of promoter | For cloning of promoter |
| Nested adapter primer* | ACTATAGGGCACGCGTGGT | Nested adapter primer 2 | For cloning of promoter |
| *GWGSP1* | CCAGAACCAGCGAAAGTCACCAGAC | Gene specific primer 1 | Gene specific primer of Promoter |
| *GWGSP2* | CCAGACTGACTCACCTAGGGTTCCAAAATC | Gene specific primer 2 | Gene specific primer of Promoter |
| *GWGSP3* | AAATCCATCAGCCCCGAATTCATG | Gene specific primer 3 | Gene specific primer of Promoter |
| *GWGSP4* | TGTCAGACATAAAATTGCCAAGAAAAGA | Gene specific primer 4 | Gene specific primer of Promoter |
