# Supplementary material for: Overexpression of WsSGTL1 Gene of Withania somnifera Enhances Salt Tolerance, Heat Tolerance and Cold Acclimation Ability in Transgenic Arabidopsis Plants
Source: PLoS One. 2013 Apr 30;8(4):e63064. doi: 10.1371/journal.pone.0063064 (PMC3639950; doi:10.1371/journal.pone.0063064)
Supplement: Table S2 — Effects of different abiotic stress on both genotypes of A. thaliana (WT and WsSGTL1 transgenic lines) in reference to fluorescence parameters. Maximum quantum yield of PSII photochemistry (Fv/Fm), effective quantum yield of PSII Y (II)), total heat dissipation (NPQ), yield of regulated heat dissipation Y (NPQ), and yield of unregulated heat dissipation Y(NO). (DOCX) [file pone.0063064.s015.docx]

**Table S2. Effect of different abiotic stresses on WT and *WsSGTL1* transgenic lines of *A. thaliana*.**

|  | **Control** | | **Salt (50 mM)** | | **Salt (100 mM)** | | **Heat (42^o^C)** | | **Cold (4^o^C)** | |
| --- | --- | --- | --- | --- | --- | --- | --- | --- | --- | --- |
|  | **Col-0** | ***SGTL1*** | **Col-0** | ***SGTL1*** | **Col-0** | ***SGTL1*** | **Col-0** | ***SGTL1*** | **Col-0** | ***SGTL1*** |
| **Fv/Fm** | 0.74 ± 0.01 | 0.72 ± 0.02 | 0.45± 0.13 | 0.70 ± 0.02 | 0.42± 0.05 | 0.59 ± 0.10 | 0.70± 0.02 | 0.75 ± 0.03 | 0.73± 0.02 | 0.72 ± 0.03 |
| **Yield** | 0.50 ± 0.02 | 0.42 ± 0.02 | 0.19± 0.08 | 0.36 ± 0.04 | 0.16± 0.04 | 0.26 ± 0.09 | 0.34± 0.06 | 0.47 ± 0.02 | 0.45± 0.03 | 0.45± 0.03 |
| **Y(NPQ)** | 0.12 ± 0.02 | 0.16 ± 0.02 | 0.09 ± 0.02 | 0.18 ± 0.02 | 0.09 ± 0.06 | 0.14 ± 0.02 | 0.10 ± 0.01 | 0.15 ± 0.01 | 0.17 ± 0.02 | 0.16 ± 0.02 |
| **Y(NO)** | 0.38 ± 0.02 | 0.41 ± 0.03 | 0.73 ± 0.10 | 0.45 ± 0.04 | 0.79 ± 0.07 | 0.60 ± 0.11 | 0.56 ± 0.06 | 0.38 ± 0.02 | 0.37 ± 0.03 | 0.38 ± 0.03 |
| **NPQ** | 0.08 ± 0.01 | 0.10 ± 0.02 | 0.03 ± 0.01 | 0.10 ± 0.02 | 0.04 ± 0.01 | 0.06 ± 0.02 | 0.04 ± 0.01 | 0.09 ± 0.01 | 0.12 ± 0.02 | 0.11 ± 0.02 |

Effect of abiotic stress with reference to fluorescence parameters. Maximum quantum yield of PSII photochemistry (Fv/Fm), effective quantum yield of PSII Y (II)), total heat dissipation (NPQ), yield of regulated heat dissipation Y (NPQ), and yield of unregulated heat dissipation Y (NO).
